# Facile Projection of Spatially Resolved Refractive Index Modulation in Monolayer MoS_2_ via Light Phase Changes

**DOI:** 10.1002/smll.202501998

**Published:** 2025-04-14

**Authors:** Yoojoong Han, Moonsang Lee, Seok Joon Yun, Ju Young Kim, Goohwan Kim, Humberto R. Gutiérrez, Hyungbin Son, Un Jeong Kim

**Affiliations:** ^1^ School of Integrative Engineering Chung‐Ang University 84, Heukseok‐ro Dongjak‐gu Seoul 06974 Republic of Korea; ^2^ Department of Materials Science and Engineering Inha University 100, Inha‐ro Michuhol‐gu Incheon 22212 Republic of Korea; ^3^ Program in Semiconductor Convergence Inha University 100, Inha‐ro Michuhol‐gu Incheon 22212 Republic of Korea; ^4^ Department of Semiconductor Physics and Engineering University of Ulsan 93, Daehak‐ro Nam‐gu Ulsan 44610 Republic of Korea; ^5^ Department of Physics University of South Florida 4202 E Fowler Ave Tampa Florida 33620 USA; ^6^ Department of Physics Dongguk University 30, Pildong‐ro 1‐gil Jung‐gu Seoul 04620 Republic of Korea

**Keywords:** band structure modulation, extinction coefficient, hyperspectral phase microscopy, refractive index, transition metal dichalcogenides

## Abstract

Fast spatial contouring of the complex refractive index (*n*  +  *ik*) of semiconducting materials is a much sought‐after goal since the advent of semiconductor‐related industries. This study develops a novel metrology to shape the refractive index modulation of materials using hyperspectral phase microscopy by maximizing the light‐matter interaction of physical properties. The facile, non‐destructive, and wide‐field hyperspectral phase technique realizes efficient visualization of the spatially resolved refractive index nature induced by strain within and among examined MoS_2_ materials. Furthermore, numerical analyses based on a steady‐state transfer matrix clarify that the spectral phase difference (Δ*ϕ*) is selectively sensitive to the modulation of refractive index (*n*) but not of extinction coefficient (*k*) under certain wavelength ranges. This dependence is associated with wavelength and the thickness of the dielectric layer on the substrates. Simple linear relation between *n* and Δ*ϕ* for ≈100 nm of SiO_2_, dielectric material supporting MoS_2_, enables to visualize the excitonic A and B band modulation, and furthermore, refractive index with fairly high precision (*coefficient of determination, R* ^2^ > 0.97 in the wavelength range of 530–630 nm).

## Graphical abstract

The modulation of refractive index (*n*) is visualized by using hyperspectral phase microscopy, where light‐matter interaction is maximized through substrate engineering. In specific wavelengths and oxide thickness of the substrate, the phase difference can directly reflect the refractive index. Furthermore, a spatially resolved refractive index distribution map of MoS_2_ is acquired without theoretical calculations. MoS_2_ samples in which strain is induced are examined by hyperspectral phase microscopy and conventional spectroscopy.

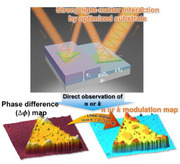

## 1 Introduction

Facile contouring of the electronic band structure in semiconducting materials is highly valued in the semiconductor industry, as it reveals essential details such as conductivity, bandgap, and the effective mass of charge carriers—factors that profoundly impact the performance of semiconductor devices. Real‐time contouring has been achieved using various advanced techniques such as angle‐resolved photoemission spectroscopy (ARPES), scanning tunneling microscopy (STM), and various computational methods.^[^ ^1^, ^2^, ^3^, ^4^, ^5^ ^]^ While these methods provide direct insights, they are typically confined to very small areas of the sample and are time‐consuming. Indirect and complementary approaches, such as Raman spectroscopy, photoluminescence (PL), transmission/reflectance, and absorption techniques, offer valuable means to contour the electronic properties of nanomaterials; however, these also exhibit limitations, such as the high‐throughput contouring of the electronic properties for large area and multilayered materials.^[^ ^6^, ^7^, ^8^, ^9^ ^]^

Recently, hyperspectral phase microscopy (HPM)—which utilizes phase‐shifting interferometry in conjunction with a Kramers–Kronig‐consistent model featuring Lorentz oscillators—has achieved notable success in wide‐field imaging of the spatially resolved complex refractive index (*n*  +  *ik*) in 2D nanomaterials on SiO_2_/Si substrates.^[^ ^10^ ^]^ HPM analysis is performed in three stages: first, the spectral phase contrast is extracted from phase images taken at various wavelengths; second, this contrast is fitted against the theoretical spectral phase contrast derived from a complex refractive index modeled by Lorentz oscillators; and third, the resulting data are used to construct images of the refractive index (*n*) or extinction coefficient (*k*). While this technique offers rapid, wide‐field imaging containing rich spectroscopic data, it faces limitations in the stability of the physical model in which Kramers–Kronig consistency is needed and two variables are estimated from one measured parameter (i.e., phase difference, Δ*ϕ*).

Strain in low‐dimensional materials has been extensively investigated as one of the facile methods for bandgap engineering, such as doping, alloying, and vertical or lateral heterostructure.^[^ ^11^, ^12^, ^13^, ^14^ ^]^ Studies have investigated various strain‐engineering methods considering the deformation of flexible substrates,^[^ ^15^, ^16^ ^]^ tip indentation,^[^ ^17^ ^]^ piezoelectric substrate,^[^ ^18^ ^]^ substrate thermal expansion,^[^ ^19^ ^]^ and blisters.^[^ ^20^ ^]^ Strain, however, can also be induced unintentionally in 2D materials grown on various supporting substrates during synthesis.^[^ ^21^ ^]^ 2D materials that are one‐atom thick can be vulnerable to extrinsic and intrinsic origins of strain, which can considerably affect their electronic band structure. Such modulation of electronic band structure induces changes of optical properties (e.g., complex dielectric functions), which can affect the physical properties of electronic and optoelectronic devices using 2D materials. Despite the importance of understanding the material's properties, a comprehensive and non‐destructive method that provides spatial information on a large scale with high throughput has not yet been developed.

In this study, we introduce an innovative system of measurement that leverages HPM to visualize the refractive index modulation of nanomaterials by optimizing light‐matter interactions. We utilized numerical analysis using the steady‐state transfer matrix^[^ ^22^ ^]^ to examine the spectral phase contrast at various wavelengths for varying thicknesses of SiO_2_ in a SiO_2_/Si substrate. Results revealed that the spectral phase contrast is selectively sensitive to the refractive index (*n*) but not the extinction coefficient (*k*), within certain wavelength ranges at a given thickness of SiO_2_. We selected an optimal oxide thickness for imaging the refractive index variation near the excitonic transitions of MoS_2_. In this condition, one unknown parameter, *n*, can be easily obtained by one measurement of phase difference (Δ*ϕ*) at a given wavelength. The modulation of A and B excitonic band frequency by strain was successfully observed via the imaging technique using MoS_2_ samples, which were prepared using four different methods; the results were consistent with photoluminescence (PL) and Raman spectroscopy. This method can be utilized to visualize refractive index modulation directly without the help of theoretical fitting to the spatially resolved phase difference data to complement current technology.

## 2 Results and Discussion

### 2.1 Strain‐Engineered Electronic Band Structure Modulation

**Figure 1:**
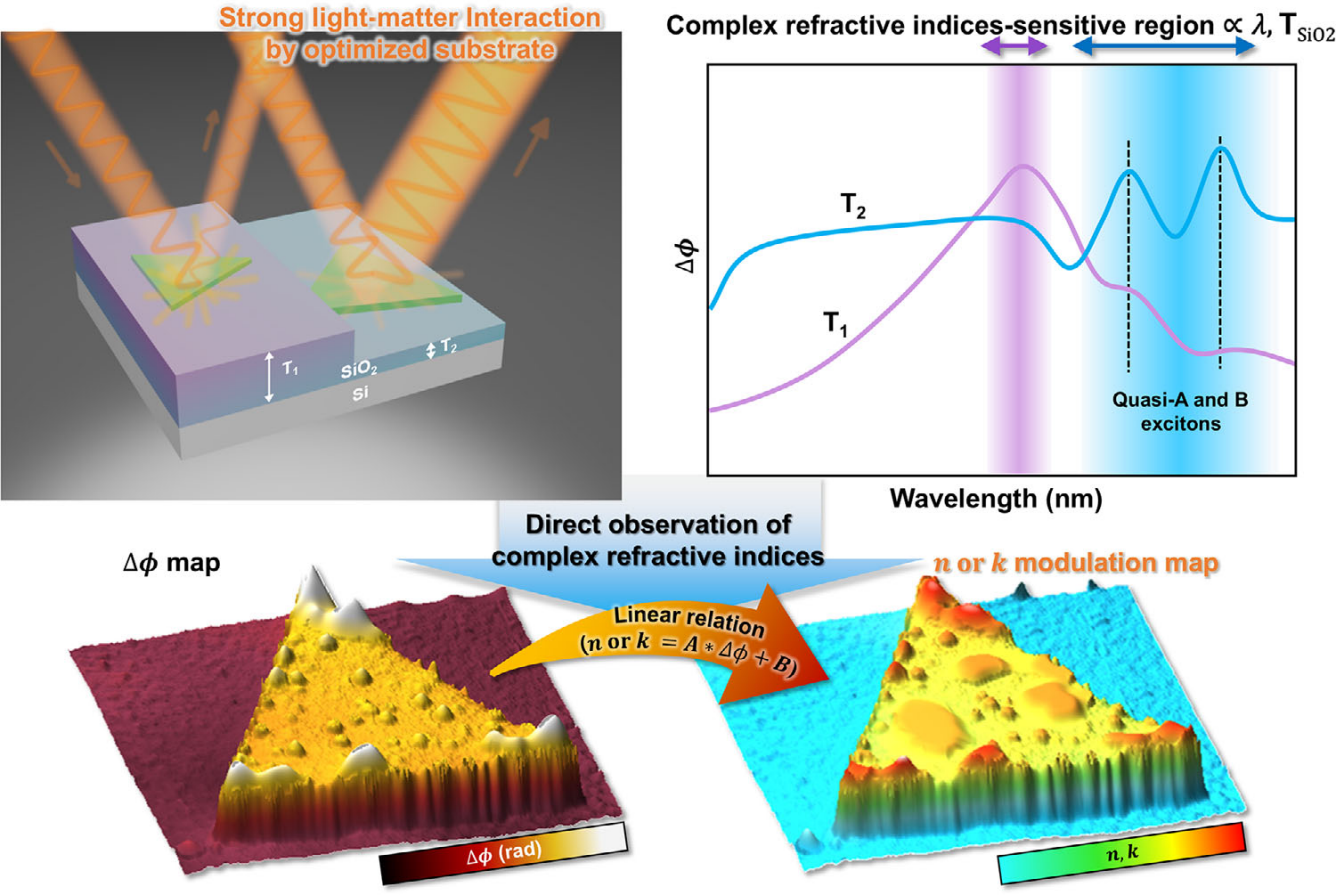
Schematic diagram of direct observation of complex refractive index through optimization of light‐matter interaction using substrate engineering, and spatially resolved complex refractive index imaging at a fixed wavelength of light using the linear relation, *n*  =  A*Δ*ϕ* + *B*.

**Figure** **1** represents a brief scheme of the process of HPM to extract complex refractive indices without theoretical fitting. The key point, herein, is the optimization of experimental conditions such as oxide thickness (T_SiO2_) and wavelength (λ) to maximize light‐matter interaction. Through substrate‐engineering, HPM enables to directly observe the complex refractive indices since phase difference (Δ*ϕ*) is linearly proportional to complex refractive indices in a certain range of wavelength (i.e., complex refractive indices‐sensitive region). Especially, the regions where the excitonic features (A and B excitons) of supported materials on the substrate exist were focused to observe the changes induced by electronic band structure modulations. We named fluctuations of phase difference by excitonic features to quasi‐A and B excitons, and underlying physics will be elucidated later.

Figure S1a (Supporting Information) shows a schematic of the strain‐engineered band structure modulation of 2D materials supported on the substrate. Several factors, such as defects, lattice mismatch, and a difference in the thermal expansion coefficient between the 2D materials and growth substrate induce local variation of strain, particularly tensile and compressive strain, within the sample.^[^ ^19^, ^23^ ^]^ A previous research on photoluminescence analysis confirmed that tensile and compressive strain cause red‐ and blueshift in the excitonic bands, respectively, leading to changes in the dielectric functions (*n*, *k*) of the material.^[^ ^8^ ^]^ Strain can be induced onto 2D materials during sample preparation, specifically in the synthesis and transfer steps, and during device fabrication. Previous research has investigated spatial modulation of dielectric functions of chemical vapor deposition (CVD)‐grown MoS_2_ using HPM.^[^ ^10^ ^]^ This technique, based on phase‐shifting interferometry, optically measures the phase difference (Δ*ϕ*  = *ϕ*_1_  − *ϕ*_0_), where *ϕ*_0_ and *ϕ*_1_ denote the final phases of light reflected by multiple interference at the reference (substrate) and sample (2D/substrate). By varying the incident wavelength, spectroscopic information of the phase difference (Δ*ϕ*) of the material can be acquired. Imaging Δ*ϕ* as a function of the incident wavelength yields a series of images that are analogous to the demosaiced images of the hyperspectral image as shown in Figure S1b (Supporting Information).^[^ ^24^, ^25^ ^]^ Changes in 2D materials by strain, doping, etc. can induce the changes in excitonic bands such as A and B excitonic band shift and oscillator strength resulting in changes of complex refractive index. Thus, by measuring Δ*ϕ* in 2D materials, spatially resolved complex refractive index variations can be monitored. A plot of Δ*ϕ* at the same local position from the data cube yields a local spectrum of the phase difference (Δ*ϕ*(λ)) that is influenced by local strain. The red and blue curves in the schematic spectra shown in Figure S1b (Supporting Information) indicate the locations of tensile and compressive strain in the sample, indicating that Δ*ϕ* is a function of the dielectric constants (*n*, *k*) and the wavelength of light (λ). Furthermore, by using the optimal substrate for the maximized light‐matter interaction, spatially resolved refractive index modulation can be easily imaged as shown in Figure 1. This study introduces a detailed strategy used to visualize the refractive index variation within and among 2D materials by light‐matter interaction.

### 2.2 Optimization of Substrate for Observing Refractive Index Modulation by HPM

**Figure 2:**
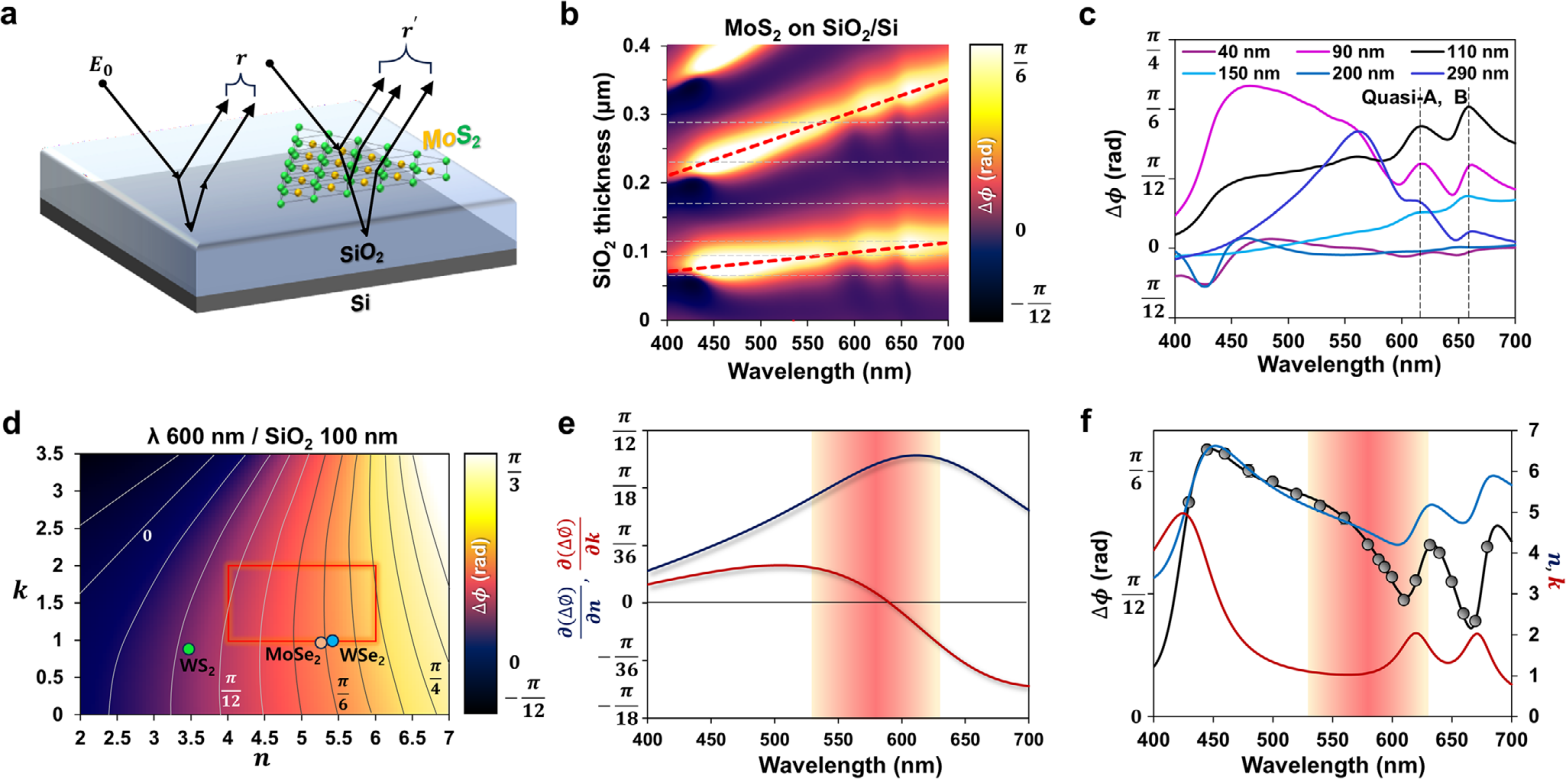
Theoretical study for optimization of substrate engineering. a) Schematic diagram of reflected light with multiple interference from supporting substrate (*r*) and MoS_2_ (*r*′). b) SiO_2_ thickness and wavelength‐dependent contour maps of the phase difference of MoS_2_/SiO_2_/Si. c) Calculated phase difference profiles of MoS_2_ as a function of wavelength with dependence on the SiO_2_ thickness in the 40–290 nm range. d) Complex refractive indices (*n*, *k*)‐dependent phase difference contour map of MoS_2_ supported on a 100 nm thick SiO_2_/Si substrate at incident light at 600 nm. The red outline box indicates a rough range of complex refractive indices of MoS_2_, where the real part (*n*) varies 4–6 and the imaginary part (*k*) 1–2 in the range of wavelength 500–700 nm. e) Partial derivative profiles of phase difference to complex refractive indices as a function of wavelength. f) Measured phase difference profiles (gray dots) with fitting (black solid lines) at 98 nm thick SiO_2_, calculated profiles of *n* (red) and *k* (blue). The *n*‐sensitive regions were pink shaded in (e,f).

**Figure** **2**a shows schematics of multiple interferences for a bare SiO_2_/Si substrate (reference) and the 2D material supported on the SiO_2_/Si substrate (sample). To understand the wavelength‐dependent phase difference, we defined the phase difference as Δ*ϕ*  = *ϕ*_1_  − *ϕ*_0_, where *ϕ*_0_ and *ϕ*_1_ denote the final phase of the light reflected by multiple interference at the reference (SiO_2_/Si) (*r*) and sample (2D/SiO_2_/Si) (*r*′), respectively. The reflected light can be expressed using the relation *r*  =  *Ae* ^*i*(ω*t* − *ϕ*)^. Thus, the final phase of light at SiO_2_/Si and 2D/SiO_2_/Si can be defined by *ϕ*_0_ = *Arg*(*r*) and *ϕ*_1_ = *Arg*(*r*′), respectively. Prior to the measurement and numerical analysis, we considered the effect of SiO_2_/Si substrate roughness, which could influence the results, as shown in Figure S2a (Supporting Information). The RMS roughness of SiO_2_/Si substrate was measured to be ≈3% of the phase difference of monolayer MoS_2_ (Δ*ϕ*  = *ϕ*_1_  − *ϕ*_0_). This indicates that the effect of substrate roughness during HPM measurement and numerical analysis is negligible. Moreover, the phase difference of monolayer MoS_2_, amplified by optical properties (e.g., refractive index) difference, represents superior contrast compared to physically measured height (0.62 nm) by atomic force microscopy (AFM) (Figure S2b, Supporting Information). A contour map for Δ*ϕ* as a function of the SiO_2_ thickness (*t*_*sio*2_) and wavelength (λ) of incident light was constructed in Figure 2b; four layers (air, monolayer MoS_2_, SiO_2_, and Si) were considered for the calculation, which was performed using the transfer matrix method.^[^ ^22^ ^]^ Figure S3 (Supporting Information) illustrates the steady‐state transfer matrix method‐based calculation which considers Fabry–Pérot interferences that occurred by multiple reflections between the layers. The optical constants of the monolayer MoS_2_ and SiO_2_/Si substrates were referenced from the literature.^[^ ^26^, ^27^, ^28^ ^]^ The result of the three‐layer (air, SiO_2,_ and Si) calculation without MoS_2_ is presented in Figure S4a (Supporting Information). The final map for the normalized electric field intensity as a function of *t*_*sio*2_ and λ agrees well with those of previous studies.^[^ ^29^ ^]^ A thinner SiO_2_ layer generally exhibits less |*E*|^2^/|*E*_0_|^2^ dependence on λ. Slopes marked by red dashed lines along the maxima of Δ*ϕ* in Figure 2b are ≈0.16 and 0.5 at the lower and higher thickness of SiO_2_, respectively. The ideal scenario, in which the normalized electric field is independent of the wavelength, presents itself when the 2D material is supported on the wedged SiO_2_ structure with a slope of 0.16; in this scenario, the spectroscopic optical properties of the supported nanomaterials can be directly obtained. However, to obtain *n* and *k* information directly, the supported material must be physically uniform, and the areal information of the supported sample cannot be determined by using the wedged SiO_2_ structure. Figure S4a (Supporting Information) shows that ≈100 nm thick layer of SiO_2_ exhibited the least dependence on λ in terms of |*E*|^2^/|*E*_0_|^2^ because ≈100 nm thick layer of SiO_2_ on Si had a lower reflectance (higher absorbance) and lesser λ‐dependence than a 290 nm thick layer (Figure S4b,c, Supporting Information). The intensity of the electric field at the SiO_2_ surface determines the strength of the light‐matter interaction between the incident light and the supported material. We determined that the extra absorbance (RSiO2(t)/Si– Rsi) of the ≈100 nm thick SiO_2_ was relatively high, in the range of 400–700 nm, and the resonance effect was weaker than that of other SiO_2_ thicknesses (e.g., 290 nm), where RSiO2(t)/Si is the reflectance from SiO_2_ /Si of with thickness t of SiO_2_ on Si substrate and Rsi is the reflectance from bare Si substrate. This trend indicates that the phase difference will be more strongly affected by MoS_2_ than SiO_2_ over the entire wavelength of light. The substructure in the range of 550–650 nm in Figure 2b originated from the wavelength‐dependent dielectric functions (*n*, *k*) of MoS_2_. Interpretation of this area proves essential for the strain‐engineered electronic band structure of MoS_2_. Figure 2c shows a plot of the phase difference (Δ*ϕ*) as a function of wavelength for various SiO_2_ thicknesses, which can be obtained by slicing the contour map at the specific thickness of SiO_2_ in Figure 2b (as indicated by the gray horizontal dashed lines). The spectra with *t*_*sio*2_ = 90 and 110 nm clearly exhibited an intrinsic shape that originated from an excitonic transition in the 550–700 nm range, as shown in Figure 2c. For thicknesses below 90 nm and above 110 nm, the same region showed unclear features owing to lower light‐matter interaction. Figure 2d shows a contour map of Δ*ϕ* as a function of *n* and *k* for visualizing the resolving power of Δ*ϕ* in response to changes in *n* and *k* of the monolayer MoS_2_; for the plot, *t*_*sio*2_ and λ were set to 100 and 600 nm, respectively. The gray solid lines are marked at every $\frac{1}{36}\pi$‐interval. The red outline box in Figure 2d serves as an eye guide for the rough range of complex refractive indices of MoS_2_ in the range of wavelength 500–700 nm. In this rough range where *n* varies 4–6 and *k* varies 1–2, *n* profiles as a function of Δ*ϕ* exhibit closed linear relation with R^2^ > 0.97 in the range of wavelength 540–630 nm with using 100 nm thick of SiO_2_ as shown in Figure S5 (Supporting Information). In other words, the 100 nm thick SiO_2_ substrate exhibited a high resolving power of Δ*ϕ* and, in response, Δ*ϕ* reflects the infinitesimal change in *n*. Additionally, Figures S6 and S7 (Supporting Information) show contour maps containing various wavelengths and SiO_2_ thicknesses, each of which displays varying dependencies on *n* and *k*. For example, for a SiO_2_ thickness of 150 nm and λ of 600 nm, Δ*ϕ* was dependent on both *n* and *k*. For a SiO_2_ thickness of 40 nm and an identical λ, Δ*ϕ* showed a strong dependence on *k*, however the resolving power for *k* is much lower than *n*‐sensitive condition. Thus we prioritized the *n*‐sensitive region for phase difference‐based analysis in this study. In Figure 2d and Figure S6 (Supporting Information), each colored dot represents the *n* and *k* values of different kinds of transition metal dichalcogenides (TMDs, e.g., MoSe_2_, WS_2_ and WSe_2_) at each wavelength.^[^ ^26^ ^]^

To investigate the sensitivity of Δ*ϕ* to *n* and *k*, we calculated the partial derivative ($\frac{\partial (\Delta \phi )}{\partial n},\frac{\partial (\Delta \phi )}{\partial k}$) as a function of wavelength, where *n*  =  5, *k*  =  1.5, and *t*_*sio*2_ = 100 nm, as shown in Figure 2e. As previously stated, a higher value of $\frac{\partial (\Delta \phi )}{\partial n}$ or $\frac{\partial (\Delta \phi )}{\partial k}$ at a specific wavelength implies a large sensitivity of Δ*ϕ* to the modulation of *n* or *k*. We defined “*n*‐sensitive” region as the range where $|\frac{\partial (\Delta \phi )}{\partial n}\left|/\right|\frac{\partial (\Delta \phi )}{\partial k}|>3$. In the case of the 100 nm thick SiO_2_ substrate, the partial derivative of *n*, ($\frac{\partial (\Delta \phi )}{\partial n}$), reached its maximum value at 610 nm with ≈100 nm span of *n*‐sensitive region (as indicated by the pink shaded area in Figure 2e), while the partial derivative of *k*, $\frac{\partial (\Delta \phi )}{\partial k}$, had a zero‐crossing at 590 nm. Table S1 (Supporting Information) illustrates the wavelength of *n*‐sensitive region and the wavelength at which the sensitivity to *k* is zero ($\frac{\partial (\Delta \phi )}{\partial k}=0$) for various thickness of the SiO_2_ substrate, where MoS_2_ are used as the supported material. Figure S8 (Supporting Information) shows contour maps for substrates of varying SiO_2_ thicknesses at λ = 600 nm with partial derivative spectra. A substrate with a 40 nm thick oxide layer shows that the partial derivative $\frac{\partial (\Delta \phi )}{\partial n}$ was nearly zero at a wavelength over 500 nm, while $\frac{\partial (\Delta \phi )}{\partial k}$ had non‐zero values over the entire wavelength range, indicating that Δ*ϕ* had a sensitivity to not *n*, but *k*. Remarkably, monolayer TMDs supported on a 110 nm thick SiO_2_ substrate exhibited *n*‐sensitive region in the near‐IR range where the electronic band transition of TMDs exists. For SiO_2_ thicknesses ranging from 200 to 300 nm, Δ*ϕ* exhibited a high sensitivity to *n* only in a narrow (less than 50 nm) range of the visible light, as indicated in Figure S8k,l (Supporting Information). Figure 2f shows Δ*ϕ* for CVD‐grown MoS_2_ supported on a 98 nm thick substrate, measured using HPM. The phase difference Δ*ϕ* was measured at 20 points in the wavelength range of 430–680 nm (gray dots). An intrinsic shape induced by excitonic transitions of monolayer MoS_2_ was clearly observed for the substrate thickness of 98 nm. Complex refractive indices (*n*, *k*) were calculated using the multiple Lorentz oscillators model (blue and red solid lines).^[^ ^10^ ^]^ The extracted *n* values resembled the Δ*ϕ* spectrum shown in Figure 2f, as expected from Figure 2d,e. The extracted *k* spectrum exhibited the A and B excitonic transition bands; however, the peak position for *n* differed slightly from that of *k*. For convenience purposes, we will refer to the two peaks in the *n*‐spectrum as quasi‐A and B excitonic transition bands.

A previous study investigated the maximizing of light outcoupling to enhance the Raman or PL signal of 2D materials by optimizing the SiO_2_ thickness and hybrid reflector.^[^ ^30^, ^31^, ^32^ ^]^ In the current work, uniform and high light outcoupling was pursued for a wide range of incident light wavelengths. Information of wavelength where phase difference directly reflects *n* in specific thickness of substrate as indicated in Table S1 (Supporting Information) can be referred to before the determination of experimental conditions which maximizes light‐outcoupling. Moreover, with the linear relationship between *n* and phase difference, i.e., *n*  =  A*Δ*ϕ* + *B* where *A* and *B* are constants, *n* of TMDs could be predicted simply without theoretical fitting model. Table S2 (Supporting Information) represents the constants A and B, and R^2^ values of linear regression between *n* and phase difference with *n* ranging 4–6 and *k* ranging 1–2 when 100 nm thick of SiO_2_ is used. With this linear relation, for example, spatially resolved *n* distribution map of CVD grown MoS_2_ supported on 98 nm was constructed from 589 nm HPM image without theoretical calculation as shown in Figure S9 (Supporting Information).

### 2.3 Monolayer MoS_2_ Prepared Under Various Stain Conditions and HPM Analysis

**Figure 3:**
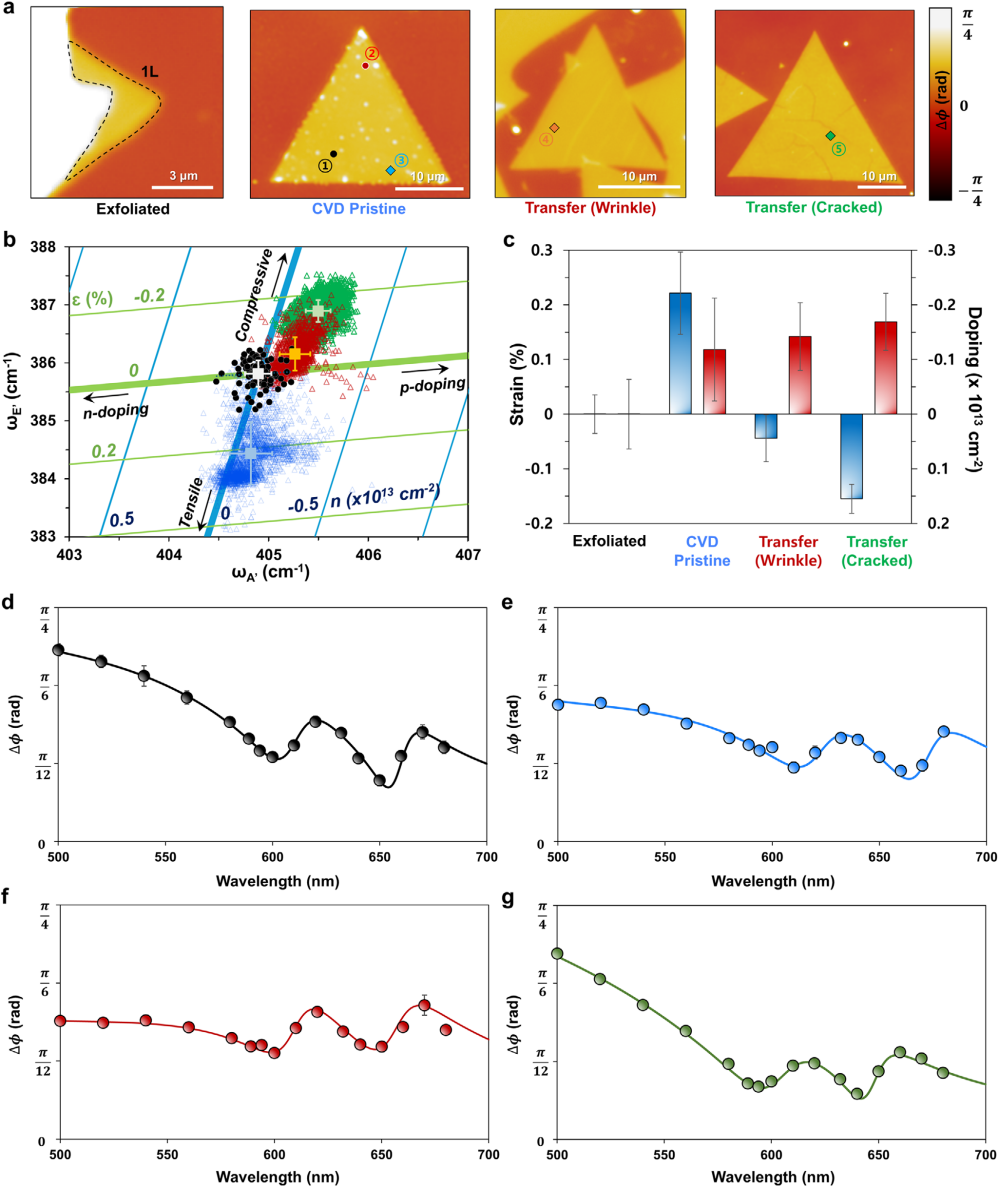
Raman and HPM analyses of strain‐engineered MoS_2_ samples. a) 560 nm phase maps of exfoliated, CVD as‐grown pristine, and transferred CVD‐grown MoS_2_ (wrinkle and cracked). b) Raman vector map representing the strain (E_2__g_) and doping (A_1__g_) of four different MoS_2_ samples (black dot: exfoliated MoS_2_, blue triangle: CVD as‐grown MoS_2_, red and green triangles: transferred CVD‐grown MoS_2_). Exfoliated MoS_2_ is referred to as strain‐ and doping‐free sample. c) Strain and doping degree histogram of each sample calculated from Raman E_2__g_ and A_1__g_ modes. d–g) Averaged phase difference profiles of each sample as a function of wavelength. Defective areas including wrinkles, tears, and speckles were excluded during averaging.

To investigate the strain‐engineered band structure modulation in monolayer MoS_2_, we prepared four different samples: exfoliated MoS_2_, CVD‐grown MoS_2_ (pristine), and two types of transferred MoS_2_ samples (wrinkled and cracked) following CVD growth (Experimental Section). The original sample preparation for wrinkled and cracked samples can be found in the literature.^[^ ^33^ ^]^ The phase difference images of the four samples at 560 nm are displayed in **Figure** **3**a. Exfoliated MoS_2_ was considered as a reference because it is both strain‐ and doping‐free.^[^ ^34^ ^]^ The phase difference images of HPM at 560 nm (Figure 3a) showed fine morphology such as speckles, wrinkles, and cracks. Raman spectroscopy and photoluminescence have been extensively utilized to understand the optical properties of 2D materials influenced by strain and doping.^[^ ^6^, ^7^, ^8^, ^10^, ^11^, ^12^, ^13^, ^14^, ^16^ ^]^ In particular, vector analysis using two Raman modes for graphene (G and 2D) and monolayer MoS_2_ (E_2__g_ and A_1__g_) have been used to quantify strain and doping in those materials.^[^ ^34^, ^35^, ^36^, ^37^ ^]^ To understand the modulation of complex refractive indies by strain and doping, Raman spectroscopy and photoluminescence have been involved for a deeper understanding the samples in this work as proactive studies. The strain and doping effects induced on the monolayer MoS_2_ can be analyzed through vector analysis using the Raman frequency of the E_2__g_ and A_1__g_ mode in monolayer MoS_2_. Using exfoliated MoS_2_ as a strain‐ and doping‐free reference (black dot), each Raman vector of the E_2__g_ and A_1__g_ frequency was constructed as shown in Figure 3b (CVD pristine indicated by blue, wrinkled MoS_2_ by red, and cracked MoS_2_ by green triangles). In each plot, the average values and standard deviation in the E_2__g_ and A_1__g_ frequency were marked, and the averaged Raman spectra of each sample are presented in Figure S10 (Supporting Information). Among the samples, strain varied in the range of ‐0.25–0.45%, while doping varied in the range of ‐0.35 × 10^13^–0 cm^−2^. Remarkably, the CVD‐grown pristine MoS_2_ sample experienced relative tensile strain, with a large strain variation (Δε∼0.56%) that is attributed to the thermal expansion mismatch between the MoS_2_ and SiO_2_ substrate.^[^ ^34^ ^]^ By contrast, transferred MoS_2_ samples experienced compressive strain with a lower deviation in strain (Δε∼0.28% and ≈0.25% in wrinkled and cracked MoS_2_, respectively), which is caused by strain relaxation during the transfer process.^[^ ^21^ ^]^ Figure 3c shows a histogram of the extracted average amounts of strain and doping with the standard deviation of each sample. Strain and doping maps reconstructed from the Raman map of each sample are presented in Figure S11 (Supporting Information). Compared to the other samples, as‐grown monolayer MoS_2_ synthesized by CVD exhibited a spatially nonuniform strain and doping degree within the sample. Figure 3d–g presents HPM profiles with guidelines, showing the phase difference as a function of wavelength (500–700 nm), which were acquired from the averaged value in the area shown in Figure 3a. Quasi‐A and B transitions of monolayer MoS_2_ were clearly observed in the HPM profile. Phase difference profiles in the range of 400–700 nm are presented in Figure S12a–d (Supporting Information). Dots imply the measured phase difference of each sample by HPM. Solid lines are the calculated phase difference values fitted using Lorentz oscillators model, and dashed lines are the 1st differential of the solid. The frequencies of A and B excitonic bands can be determined using the 1st differential. The frequencies of the A and B excitonic bands varied significantly amongst the samples. Owing to differences in SiO_2_ substrate thickness (90, 98, 100, and 85 nm), each sample exhibited a differently shaped Δ*ϕ* profile in the short wavelength. The quasi‐A and B excitonic band frequencies in the Δ*ϕ* profiles resembling the refractive index (*n*) spectrum facilitate the understanding of the refractive index modulation of MoS_2_ by strain.

**Figure 4:**
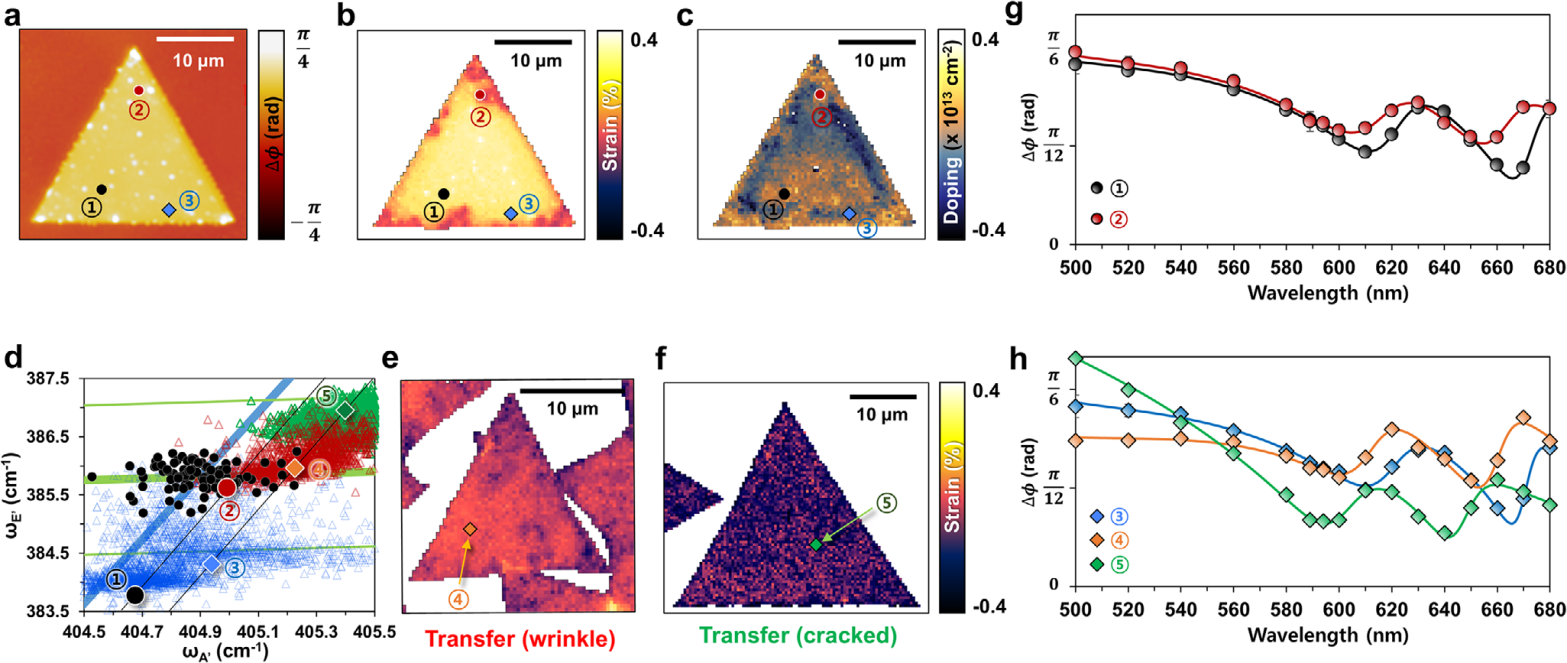
HPM analysis of strain‐induced refractive index modulation. a) 560 nm phase map, b) strain map, and c) doping map of the pristine MoS_2_ sample. d) Blown‐up Raman vector map from Figure 3b with marks of five selected points having different strain and doping. e,f) Strain maps of transferred CVD‐grown MoS_2_ samples. g,h) phase difference profiles as a function of wavelength selected from doping‐independent points of intra‐sample (g) (point ① and ② at pristine MoS_2_) and of inter‐sample (h) (point ③ at pristine MoS_2_, ④ at wrinkled, and ⑤ at cracked transferred MoS_2_, respectively).

To validate this analysis, the HPM results were directly compared for intra‐ and inter‐sample strain while excluding doping effect, as shown in **Figure** **4**. Local points indicated by ①–⑤, representing both intra‐ and inter‐sample strain, were selected using the strain‐doping vector map, as shown in Figure 4d. First, in the CVD pristine MoS_2_ sample, two points (marked as ① and ②, representing intra‐sample strain) were chosen at which the strain variation was maximized (Δε = 0.19%). Simultaneously, the doping effect was neglected for these two points (Figure 4a–c). Each point was marked on the Raman frequency plot, as shown in Figure 4d, following which they were laid on the equal doping status line presenting different strains, 0.24 and 0.05%, respectively. For the inter‐sample comparison, points ③, ④, and ⑤, presenting the same doping level, were chosen from CVD pristine, transferred MoS_2_ containing wrinkles and cracks (Figure 4a–c,e,f, respectively). Figure 4d shows the three solid diamond symbols on the line, representing equal doping levels and variations in strain, i.e., 0.21, ‐0.08, and ‐0.16%, respectively. HPM profiles of point ①/②(intra‐sample) and ③/④/⑤(inter‐sample) are shown in Figure 4g,h, respectively. Both cases exhibited prominent strain‐modulated phase difference profiles. Both A and B excitons in point ② (663 and 616 nm) were blueshifted against point ① (673 and 622 nm), where point ② experienced relative compressive strain (Figure 4g). The results agree well with those of previous research in which compressive and tensile strain‐induced blue‐ and redshift in A and B excitonic transitions by PL, respectively. Likewise, in Figure 4h, from point ③ (A and B exciton are measured by 671 and 621 nm) to point ④ (660.5 and 612 nm) and ⑤ (648.5 and 604 nm), it was confirmed that the A and B exciton experiences blueshift that are induced by compressive strain. These results agree well with PL measurement (Figure S13, Supporting Information), where A exciton of 675 nm in unstrained sample (point ⓐ) was blueshifted to 663 nm (point ⑤) and redshifted to 681 nm (point ③) by compressive and tensile strain, respectively. This result underlines the versatility of HPM in strain‐engineering applications. Besides, refractive index modulation by doping is investigated by maximizing the doping variation within the sample while the strain fixed as shown in Figure S14 (Supporting Information). The gray and pink triangles on the red solid line in Figure S14e (Supporting Information) indicate that there is no significant change in the quasi‐A and B excitonic bands in the HPM spectrum.

By optimizing the thickness of oxide layer, light‐matter interaction for wavelength can be maximized, thereby allowing phase difference to be dominated by of supported material and to simultaneously reflect its refractive (*n*). From this analysis, modulation of the quasi‐excitonic band and refractive index caused by strain, doping and etc. can be easily, non‐destructively, and promptly imaged using HPM.

### 2.4 Visualization of Strain‐Induced Modulation of Refractive Index by HPM

**Figure 5:**
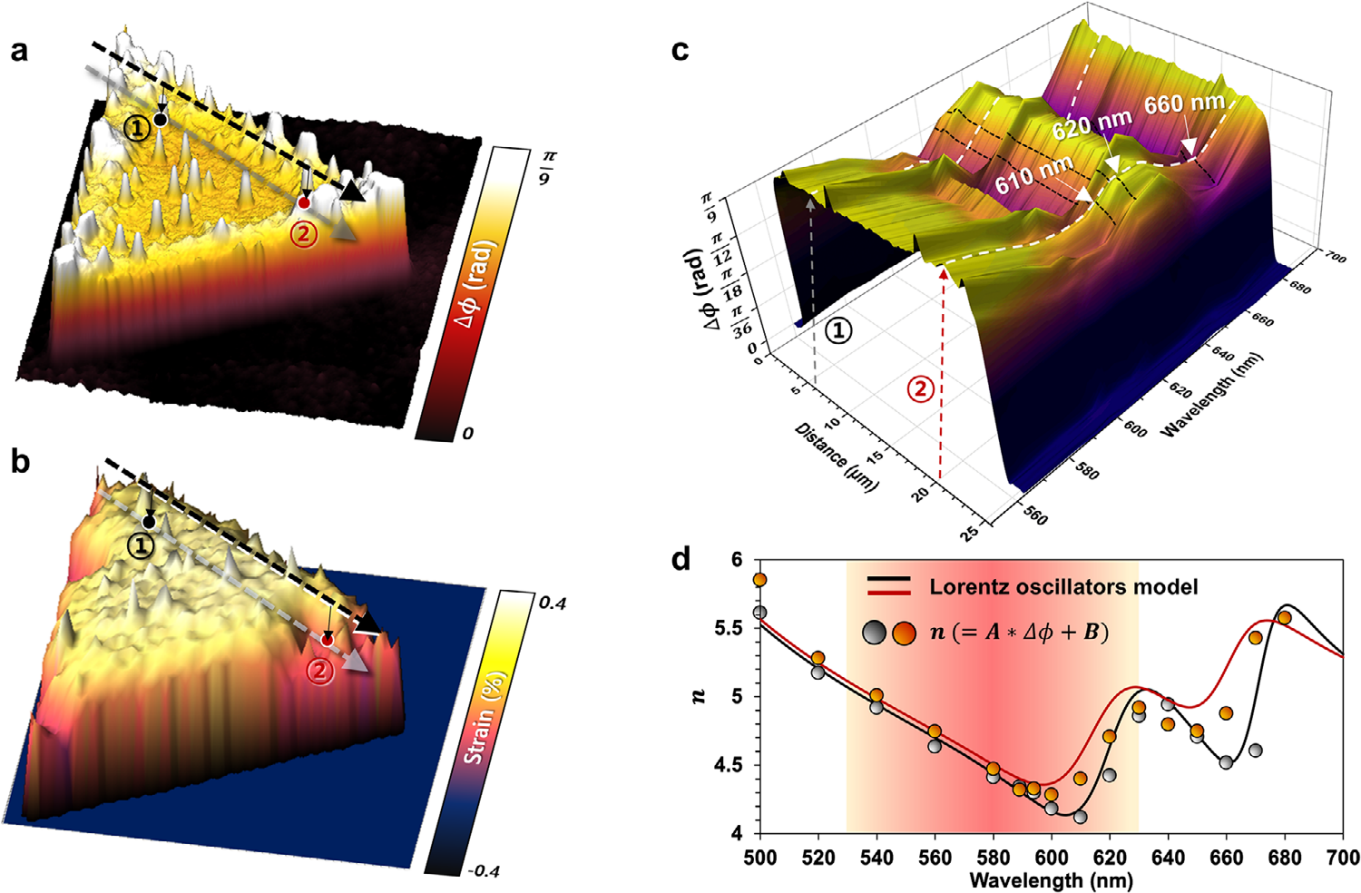
Visualization of refractive index modulation by HPM. a,b) 3D maps of phase difference at 620 nm (a) and strain of pristine sample (b). c) 3D contour map constructed by stacking line profiles of dashed arrow from (a,b) into wavelength‐direction. Doping‐independent points (① and ②) from Figure 4a are marked with dashed arrows on the maps. White arrows indicate the wavelength, which shows a relatively large phase difference modulation by strain (610, 620, and 660 nm). d) Refractive index (*n*) spectra derived from linear relation to phase difference at points ① and ② (gray and orange dots) in the range of wavelength 500–680 nm. Solid lines are refractive index (*n*) obtained by fitting to the HPM experimental data by Krammers‐Kronig consistant model with Lorentz oscillators.

HPM is a versatile tool for optical analysis offering high spatial and height resolution and can be used to visualize strain‐induced modulation of Δ*ϕ* or complex refractive indices (*n, k*) in various forms of data such as spectra (Δ*ϕ* or v wavelength, or (*n, k*) vs wavelength) and wavelength‐dependent 2D and 3D Δ*ϕ* or (*n, k*) maps, which we explain as follows. Unlike atomic force microscopy (AFM), chemical and physical changes in the sample can be visualized depending on the incident wavelength. **Figure** **5**a shows a phase difference (Δ*ϕ*) image of a CVD‐grown pristine MoS_2_ flake on a SiO_2_/Si substrate imaged using incident light at 620 nm. Particularly, HPM images of the wavelength in the range of the A and B transition (610, 620, 660, and 670 nm) clearly reflect the strain‐modulated refractive index (Figure S15, Supporting Information). Figure 5b shows the strain map in 3D obtained using Raman vector analysis, as shown in Figure 4d. The line profiles showing strain variation (Figure 5a,b) can be stacked in the direction of the wavelength, as shown in Figure 5c. This allows the variation in the refractive index to be visualized along the line within the sample. Figure 5d illustrates *n* values derived from linear relation to phase difference in the range of wavelength 500–680 nm. Gray and orange dots are from the point ① and ② indicated in Figure 5a. Solid lines are the calculated refractive index (*n*) by fitting Kramers–Kronig consistent model with oscillators to wavelength dependent HPM. As explained earlier, the pink shaded area exhibits Δ*ϕ* dominant with *n* dependence resulting in $R^{2}>∼0.97$. In this region, *n* can be determined using the linear relation *n*  =  A*Δ*ϕ* + *B*, where *A* and *B* are constants referred from Table S2 (Supporting Information). Remarkably, in *n*‐sensitive region (530–630 nm), derived *n* values are reasonably well matched to each other, where root mean square (RMS) errors are 0.104 and 0.123 at point ① and ②, respectively. However, errors at outer range of *n*‐sensitive region increased to 0.16 and 0.201. These results confirm that while Lorentz oscillator fitting remains necessary for general cases, the linear approximation provides a reasonable estimation of *n* within the identified *n*‐sensitive region. In other words, a simplified linear approximation offers a practical and time‐efficient alternative. In addition, Spatially resolved *n* maps of each wavelength were constructed from phase difference maps without theoretical calculation as shown in Figure S16 (Supporting Information). Both constructed 3D contour phase difference map and derived *n* spectra with spatially resolved maps allow the strain‐engineered modulation of excitonic transitions in specific wavelength to be easily recognized using the optimized substrate engineering without the need for complicated calculations. The development of a simple algorithm enables the acquisition and comparison of spectrum variation. Using this methodology, we were able to visualize refractive index modulation by strain on the optimized SiO_2_ thickness using HPM. By designing the oxide material, light‐matter interaction can be further optimized for a wide range of materials. In the future, this advancement, as metrology and an inspection tool, could be indispensable in the semiconductor industry by enabling more accurate characterization and quality control of nanoscale and multi‐layered devices.

## 3 Conclusion

This study demonstrated the facile projection of spatially resolved refractive index modulation of monolayer MoS_2_ by strain using HPM. The method supports substrate engineering without the need for complicated theoretical calculation to interpret phase difference (Δ*ϕ*) data. Using theoretical calculations to optimize the SiO_2_ thickness and thus maximize the light‐matter interaction, the phase difference was dominated by MoS_2_ in a wide range of the wavelength. The steady‐state transfer matrix model showed that the spectral phase difference is selectively sensitive to variations in the refractive index (*n*) but not the extinction coefficient (*k*) for a certain wavelength range at a given thickness of SiO_2_ in the SiO_2_/Si substrate. Using this methodology, we successfully observed refractive index modulation of intra‐ and inter‐sample by strain. A comparison of Raman and PL analysis underlined the versatility of HPM in strain‐engineering applications in providing material properties of the substrate in the form of a spectrum. In the refractive index (*n*) sensitive condition at ≈100 nm of SiO_2_/Si substrate, *n* is linearly proportional to Δ*ϕ* with high precision (*R* ^2^ > 0.97 in the range of 530–630 nm and *R* ^2^ > 0.9 in the range of 500–700 nm). Using this simple linear relation of *n* to Δ*ϕ*, excitonic A and B band modulation in spectrum can be obtained without complicated theoretical model. As the extinction coefficient (*k*) plays a crucial role in optical transitions and electronic structures, future studies may explore *k*‐sensitive conditions using different materials or alternative optical measurement techniques. Furthermore, spatially resolved refractive index (*n*) modulation by strain in the inter‐ and intra‐MoS_2_ samples was clearly visualized. Although strain engineering of 2D materials has made great progress, strain in 2D material cannot reach the theoretically predicted limit owing to the interfacial interaction strength betwfeen the substrate and 2D materials.^[^ ^7^, ^38^ ^]^ This technique could inform the level of strain applied to the nanomaterials until the desired level of strain. Moreover, it can be extended to other factors, such as doping, stoichiometry, and various kinds of defects, to modulate refractive index. Additionally, as the electronic band structure of 2D materials varies with the number of layers and stacking configuration (e.g., 2H and 3R, or misoriented angle), investigating these dependencies in complex refractive indices by HPM can be an interesting subject to be done in the near future. Band bending at interfaces between two different materials, and imperfections such as layer thickness, grain boundaries and any other types of defects need to be clarified how those can affect phase difference in 2D materials. By supporting substrate engineering, light‐matter interaction can be further optimized for phase difference across a wide range of materials and wavelengths (e.g., UV and infrared) with narrower spectral resolution of excitation source and continuous wavelengths, and consequently maximize the visibility of the physical property of interest. This imaging technique can be developed for on‐cell metrology and as an inspection tool for nanoscale semiconductors and multi‐layered semiconducting devices in terms of in situ and real‐time manner in the future. Furthermore, this tool can aid the development of innovative measurement systems for future fundamental research and technology.

## 4 Experimental Section

### Synthesis of Strain‐Engineered MoS_2_ Monolayer Samples

The MoS_2_ monolayer was synthesized by the CVD method using liquid metal precursors. The liquid metal precursors were prepared by mixing an aqueous solution of 0.01 m sodium molybdate dihydrate (Na_2_MoO_4_∙2H_2_O, Sigma–Aldrich, 331058) and an iodixanol solution (Sigma–Aldrich, D1556) at a ratio of 8:1. The mixed solution was spin‐coated onto the SiO_2_/Si substrate at 3000 rpm for 1 min.

The liquid precursor coated substrate together with pure solid sulfur (Sigma–Aldrich, 344621) were introduced into the two‐inch two‐zone furnace CVD chamber. For the growth of the MoS_2_ monolayer, the temperature of substrate zone was elevated to 765 °C under Ar atmosphere at a flow rate of 500 sccm, while the temperature of the sulfur zone reached 210 °C and was maintained for 10 min. Afterward, both furnaces were opened to allow the temperatures to cool naturally.

### Transfer Technique for Strain‐Engineering MoS_2_ Monolayer Samples

The as‐grown CVD MoS_2_ monolayer on the SiO_2_/Si substrate was tensile strained owing to the difference in the thermal expansion coefficients between MoS_2_ and SiO_2_. The strain can be relaxed or engineered during the conventional Polymethyl methacrylate (PMMA)‐supported wet transfer process.^[^ ^33^, ^39^ ^]^ Applied strain for the MoS_2_ monolayer was varied by adjusting the temperature of the rinsing deionized (DI) water bath during the PMMA‐supported wet transfer process. The PMMA (Microchem, 950 PMMA C4, 28‐05773‐24) was spin‐coated on the MoS_2_/SiO_2_/Si sample at 1500 rpm for 1 min. The PMMA‐supported MoS_2_ monolayer film was delaminated from the SiO_2_/Si substrate by immersing the sample in an HF solution, following which the PMMA/MoS_2_ film was transferred to a clean DI water bath to remove any undesired chemicals. The temperature of the DI water bath was lowered to 3 °C or raised to 80 °C to induce the contraction (or expansion) of the PMMA supporting layer, which induced compressive (or tensile) strain into the MoS_2_ monolayers, eventually resulting in wrinkles (or cracks). The PMMA‐supported MoS_2_ sample was then quickly transferred to an arbitrary substrate and dried in a cold (or hot) nitrogen atmosphere. Finally, the PMMA layer was removed using acetone, resulting in a wrinkled or cracked MoS_2_ monolayers on the substrate.

### Hyperspectral Phase Microscopy (HPM)

The configuration and mechanism of HPM were previously reported.^[^ ^10^ ^]^ In brief, the lab‐made instrument consists of an optical microscope (Olympus), piezoelectric stage (PI), and CMOS image sensor (XIMEA). After successively obtaining eight interferograms at an interval of *λ*/8 under ambient temperature (25 °C) and pressure conditions (1 atm), the phase image was constructed using the five‐step Schwider–Hariharan algorithm.^[^ ^40^ ^]^ The phase difference was calculated as, where and are the phase at the sample (2D/SiO_2_/Si) and reference(SiO_2_/Si), respectively. For hyperspectral measurement, a white LED source was filtered through 20 band‐pass filters with a FWHM of 10 nm (Edmund Optics) in the range of 430–680 nm with 10 to 20 nm intervals. In this experiment, very low light intensity of 1≈2 mW cm^−2^ was used for the entire wavelength range. The absorption at SiO_2_ surface varies by ∼ three times in the range of 400–700 nm as shown in Figure S3c (Supporting Information). The temperature dependence of incident wavelength of light could be ignored in the experimental condition of this work. HPM was based on phase‐shifting interferometry, and its lateral resolution was ≈500 nm when using a 50 × Mirau objective lens (Nikon, numerical aperture 0.55). This value depends on both the wavelength of light and numerical aperture of the objective lens. The vertical resolution of HPM was known as ≈0.1 nm in the literatures^[^ ^35^, ^41^ ^]^

### Confocal Raman and Photoluminescence Spectroscopy

For investigating the strain‐engineering effect of the MoS_2_ samples, confocal micro‐Raman and PL measurements were conducted using XperRAM S (NANOBASE). A 532 nm laser at an intensity of 150 µW was used as an excitation source for both Raman and PL measurements. Raman and PL mapping was conducted with 0.5 s of acquisition of each spectrum and 0.4 µm spatial resolution using a 50 × objective lens (Olympus). Gratings of 1800 and 300 lines mm^−1^ were utilized in Raman and PL experiments, respectively.

## Conflict of Interest

The authors declare no conflict of interest.

## Supporting information



Supporting Information

## Data Availability

The data that support the findings of this study are available from the corresponding author upon reasonable request.

## References

[1] S. W. Jung , S. Pak , S. Lee , S. Reimers , S. Mukherjee , P. Dudin , T. K. Kim , M. Cattelan , N. Fox , S. S. Dhesi , C. Cacho , S. N. Cha , Appl. Surf. Sci. 2020, 532, 147390.

[2] F. Liu , Chem. Sci. 2022, 14, 736. 36755720 10.1039/d2sc04124c PMC9890651

[3] P. Vancsó , G. Z. Magda , J. Peto , J. Y. Noh , Y. S. Kim , C. Hwang , L. P. Biró , L. Tapasztó , Sci. Rep. 2016, 6, 29726. 27445217 10.1038/srep29726 PMC4957227

[4] Y. Ma , R. A. Kalt , A. Stemmer , RSC Adv. 2022, 12, 24922. 36199876 10.1039/d2ra05123k PMC9434384

[5] C. Zhang , C. P. Chuu , X. Ren , M. Y. Li , L. J. Li , C. Jin , M. Y. Chou , C. K. Shih , Sci. Adv. 2017, 3, 1601459. 10.1126/sciadv.1601459 PMC5218515 28070558

[6] A. Castellanos‐Gomez , R. Roldán , E. Cappelluti , M. Buscema , F. Guinea , H. S. J. Van Der Zant , G. A. Steele , Nano Lett. 2013, 13, 5361. 24083520 10.1021/nl402875m

[7] J. Lee , S. J. Yun , C. Seo , K. Cho , T. S. Kim , G. H. An , K. Kang , H. S. Lee , J. Kim , Nano Lett. 2021, 21, 43. 33052049 10.1021/acs.nanolett.0c02619

[8] S. Pak , J. Lee , Y. W. Lee , A. R. Jang , S. Ahn , K. Y. Ma , Y. Cho , J. Hong , S. Lee , H. Y. Jeong , H. Im , H. S. Shin , S. M. Morris , S. Cha , J. I. Sohn , J. M. Kim , Nano Lett. 2017, 17, 5634. 28832158 10.1021/acs.nanolett.7b02513 PMC5959243

[9] I. Niehues , A. Blob , T. Stiehm , R. Schmidt , V. Jadriško , B. Radatović , D. Čapeta , M. Kralj , S. M. De Vasconcellos , R. Bratschitsch , 2D Mater. 2018, 5, 031003.

[10] U. J. Kim , Y. Han , F. A. Nugera , S. J. Yun , S. I. Kim , M. Lee , H. R. Gutiérrez , Y. H. Lee , H. Son , Nano Today 2024, 55, 102170.

[11] K. F. Mak , K. He , C. Lee , G. H. Lee , J. Hone , T. F. Heinz , J. Shan , Nat. Mater. 2013, 12, 207. 23202371 10.1038/nmat3505

[12] F. A. Nugera , P. K. Sahoo , Y. Xin , S. Ambardar , D. V. Voronine , U. J. Kim , Y. Han , H. Son , H. R. Gutiérrez , Small 2022, 18, 2106600. 10.1002/smll.202106600 35088542

[13] Y. Han , U. J. Kim , F. A. Nugera , S. I. Kim , Y. Park , M. Cheon , G. C. Kim , H. R. Gutiérrez , M. Lee , H. Son , Phys. Status Solidi B Basic Res. 2023, 260, 2200501.

[14] C. Cho , J. Wong , A. Taqieddin , S. Biswas , N. R. Aluru , S. Nam , H. A. Atwater , Nano Lett. 2021, 21, 3956. 33914542 10.1021/acs.nanolett.1c00724

[15] S. B. Desai , G. Seol , J. S. Kang , H. Fang , C. Battaglia , R. Kapadia , J. W. Ager , J. Guo , A. Javey , Nano Lett. 2014, 14, 4592. 24988370 10.1021/nl501638a

[16] H. J. Conley , B. Wang , J. I. Ziegler , R. F. Haglund , S. T. Pantelides , K. I. Bolotin , Nano Lett. 2013, 13, 3626. 23819588 10.1021/nl4014748

[17] S. Manzeli , A. Allain , A. Ghadimi , A. Kis , Nano Lett. 2015, 15, 5330. 26191965 10.1021/acs.nanolett.5b01689

[18] Y. Y. Hui , X. Liu , W. Jie , N. Y. Chan , J. Hao , Y. T.e Hsu , L. J. Li , W. Guo , S. P. Lau , ACS Nano 2013, 7, 7126. 23844893 10.1021/nn4024834

[19] G. Plechinger , A. Castellanos‐Gomez , M. Buscema , H. S. J. Van Der Zant , G. A. Steele , A. Kuc , T. Heine , C. Schüller , T. Korn , 2D Mater. 2015, 2, 015006.

[20] R. Yang , J. Lee , S. Ghosh , H. Tang , R. M. Sankaran , C. A. Zorman , P. X. L. Feng , Nano Lett. 2017, 17, 4568. 28628325 10.1021/acs.nanolett.7b00730

[21] S. Luo , C. P. Cullen , G. Guo , J. Zhong , G. S. Duesberg , Appl. Surf. Sci. 2020, 508, 145126.

[22] S. J. Byrnes , arXiv 2016, arXiv:160302720.

[23] M. Y. Li , Y. Shi , C. C. Cheng , L. S. Lu , Y. C. Lin , H. L. Tang , M. L. Tsai , C. W. Chu , K. H. Wei , J. H. He , W. H. Chang , K. Suenaga , L. J. Li , Science 2015, 349, 524. 26228146 10.1126/science.aab4097

[24] S. Lee , H. Kim , G. Kim , H. Son , U. J. Kim , Adv. Mater. Technol. 2025,10, 2400671.

[25] S. Lee , H. Kim , S. Kim , H. Son , J. S. Han , U. J. Kim , ACS Sens. 2024, 10, 236. 39721943 10.1021/acssensors.4c02213

[26] C. Hsu , R. Frisenda , R. Schmidt , A. Arora , S. M. de Vasconcellos , R. Bratschitsch , H. S. J. van der Zant , A. Castellanos‐Gomez , Adv. Opt. Mater. 2019, 7, 1900239.

[27] D. Franta , D. Nečas , I. Ohlídal , A. Giglia , Optical Micro‐and Nanometrology VI 2016, 9890, 989014.

[28] D. Franta , P. Franta , J. Vohánka , M. Cermák , I. Ohlídal , J. Appl. Phys. 2018, 123, 185707.

[29] X. Li , Y. Shi , S. Li , W. Shi , W. Han , C. Zhou , X. Zhao , B. Liang , Opt. Mater. Express 2018, 8, 3082.

[30] H. Y. Jeong , U. J. Kim , H. Kim , G. H. Han , H. Lee , M. S. Kim , Y. Jin , T. H. Ly , S. Y. Lee , Y. G. Roh , W. J. Joo , S. W. Hwang , Y. Park , Y. H. Lee , ACS Nano 2016, 10, 8192. 27556640 10.1021/acsnano.6b03237

[31] K. V. Sreekanth , P. Prabhathan , A. Chaturvedi , Y. Lekina , S. Han , S. Zexiang , E. H. Tong Teo , J. Teng , R. Singh , Small 2022, 18, 2202005. 10.1002/smll.202202005 35714298

[32] M. Zhang , Y. Zhou , P. Li , Z. Li , Opt. Express 2024, 32, 42569.

[33] S. J. Y. et al. in preparation.

[34] W. H. Chae , J. D. Cain , E. D. Hanson , A. A. Murthy , V. P. Dravid , Appl. Phys. Lett. 2017, 111, 143106.

[35] U. Lee , Y. S. Woo , Y. Han , H. R. Gutiérrez , U. J. Kim , H. Son , Adv. Mater. 2020, 32, 2070288. 10.1002/adma.202002854 32797695

[36] A. Michail , N. Delikoukos , J. Parthenios , C. Galiotis , K. Papagelis , Appl. Phys. Lett. 2016, 108, 173102.

[37] S. Dubey , S. Lisi , G. Nayak , F. Herziger , V. D. Nguyen , T. Le Quang , V. Cherkez , C. González , Y. J. Dappe , K. Watanabe , T. Taniguchi , L. Magaud , P. Mallet , J. Y. Veuillen , R. Arenal , L. Marty , J. Renard , N. Bendiab , J. Coraux , V. Bouchiat , ACS Nano 2017, 11, 11206. 28992415 10.1021/acsnano.7b05520

[38] F. Wang , B. Zhou , H. Sun , A. Cui , T. Jiang , L. Xu , K. Jiang , L. Shang , Z. Hu , J. Chu , Phys. Rev. B 2018, 98, 245403.

[39] X. Duan , C. Wang , Z. Fan , G. Hao , L. Kou , U. Halim , H. Li , X. Wu , Y. Wang , J. Jiang , A. Pan , Y. Huang , R. Yu , X. Duan , Nano Lett. 2016, 16, 264. 26633760 10.1021/acs.nanolett.5b03662

[40] P. Hariharan , B. F. Oreb , T. Eiju , Appl. Opt. 1987, 26, 2504. 20489904 10.1364/AO.26.002504

[41] D. K. Venkatachalam , P. Parkinson , S. Ruffell , R. G. Elliman , Appl. Phys. Lett. 2011, 99, 234106.

