# Supplementary material for: Facile Projection of Spatially Resolved Refractive Index Modulation in Monolayer MoS2 via Light Phase Changes
Source: Small. 2025 Apr 14;21(23):2501998. doi: 10.1002/smll.202501998 (PMC12160670; doi:10.1002/smll.202501998)
Supplement: Supplementary file 1 — Supporting Information [file SMLL-21-2501998-s001.docx]

Supporting Information

Facile Projection of Spatially Resolved Refractive Index Modulation in monolayer MoS_2_ via light phase changes

Yoojoong Han, Moonsang Lee, Seok Joon Yun, Ju Young Kim, Humberto R. Gutiérrez, Hyungbin Son*, Un Jeong Kim*


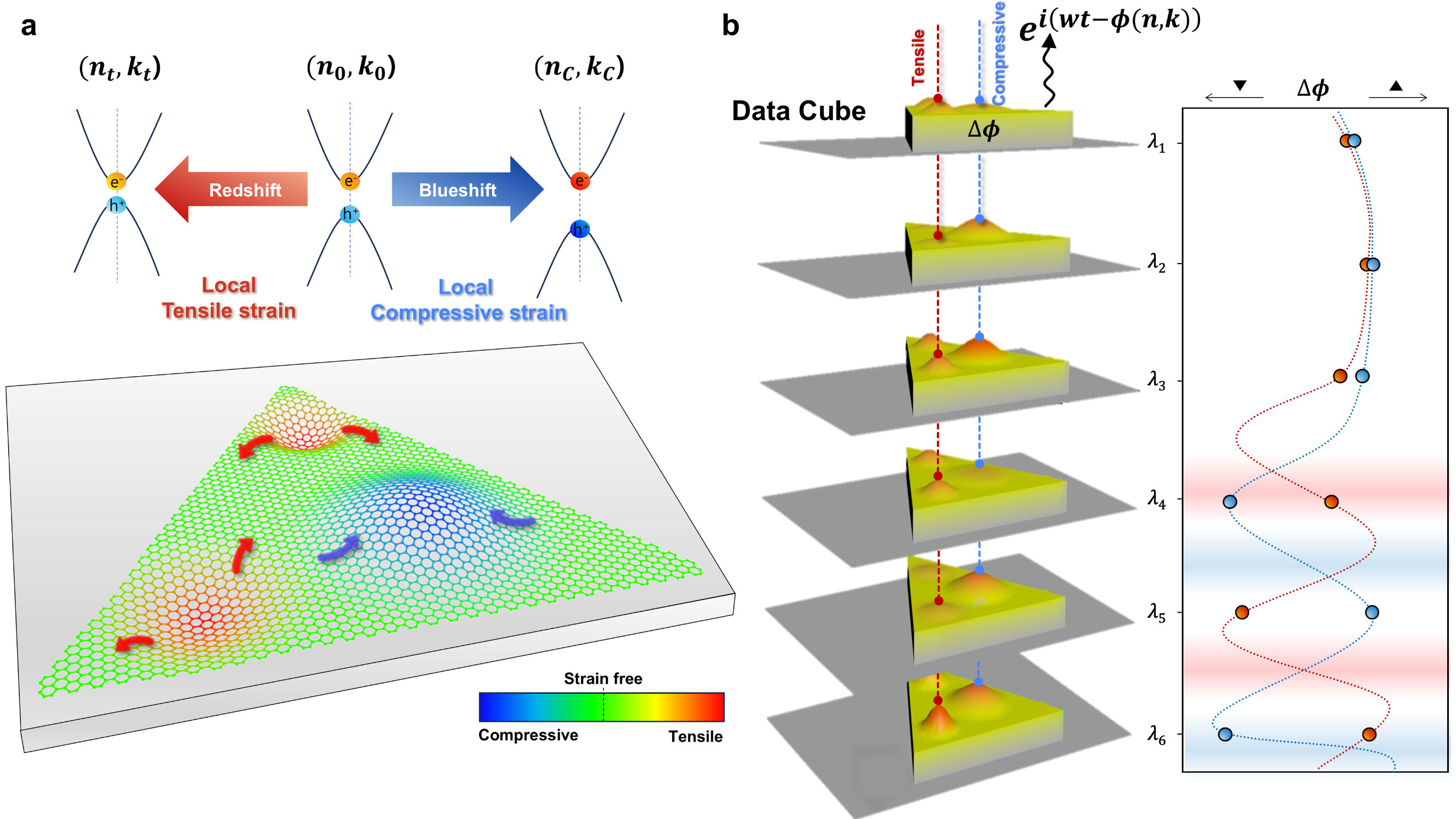


**Figure S1**. Strain-induced modulation of electronic band structure and observation using hyperspectral phase microscopy (HPM). **(a)** Schematic diagram showing the modulation of electronic band structure by strain. **(b)** Hyperspectral phase images of 2D materials at each wavelength with extracted phase difference ($\Delta\phi$) profiles.


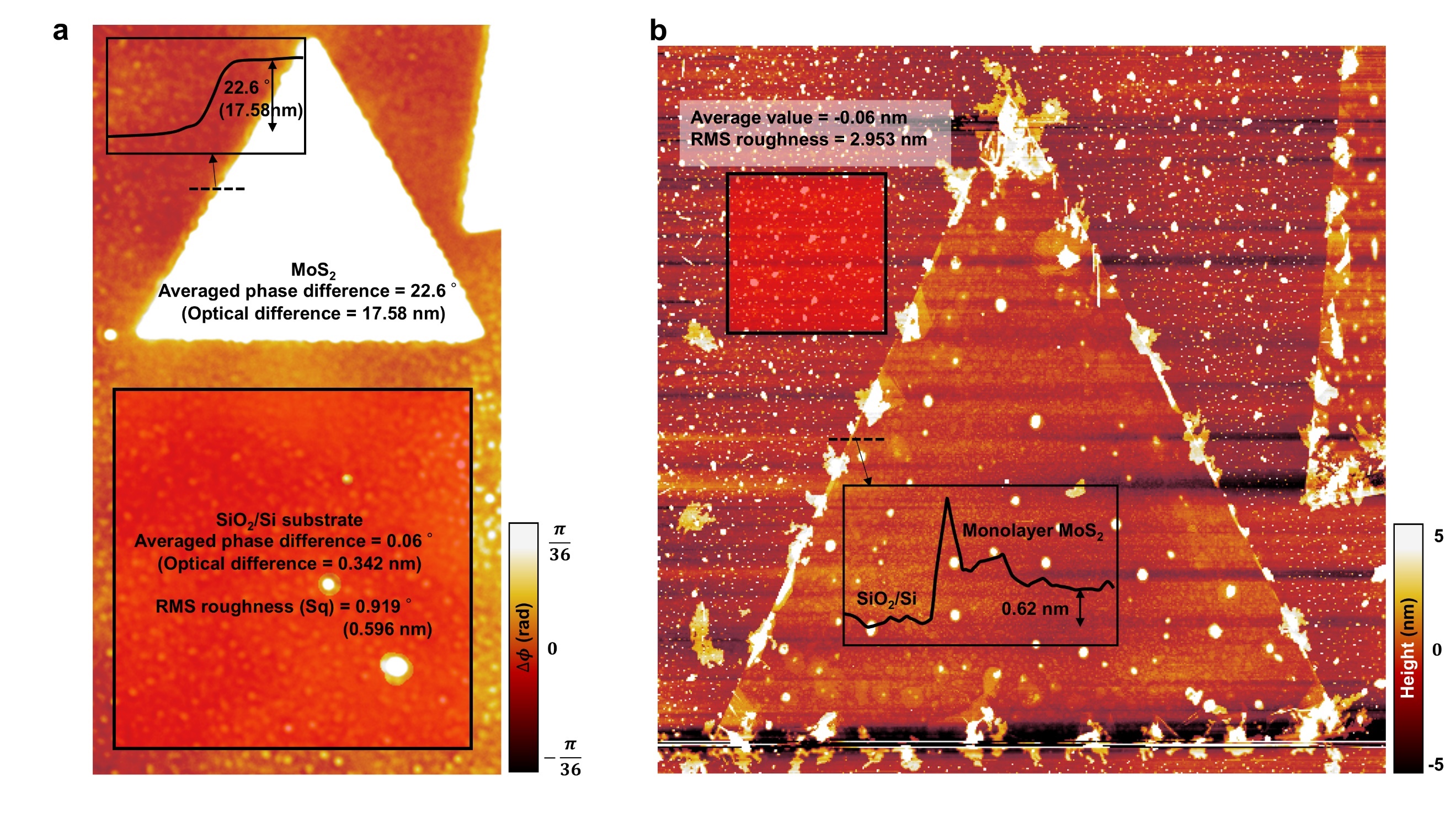
 **Figure S2**. **(a)** 560 nm HPM image of monolayer MoS_2_ supported on 98 nm SiO_2_/Si substrate. The average phase difference (optical height) and RMS roughness of the silicon substrate in the area highlighted in the red box was measured. The inset profile represents phase difference (optical height) of monolayer MoS_2_ ($\Delta\phi=\phi_{1}-\phi_{0}$). **(b)** Atomic force microscopy image of the same sample in **(a)**. Likewise, the roughness of SiO_2_/Si substrate of red-highlighted area was measured. The inset profile confirms that MoS_2_ is monolayer.


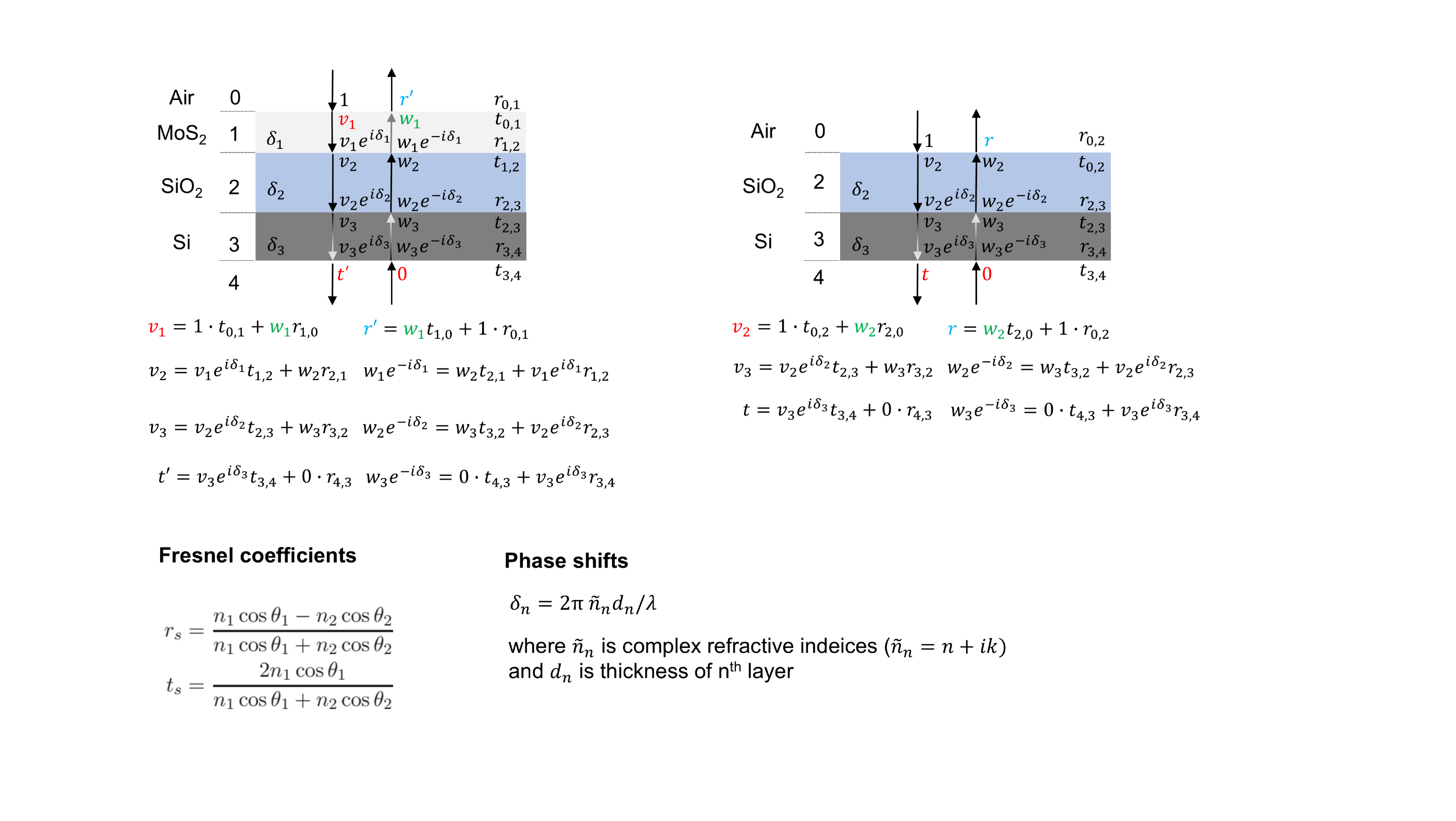


**Figure S3**. Steady-state transfer method-based calculation of phase difference. The final reflection coefficients, accounting multiple reflections ($\boldsymbol{r}^{\boldsymbol{'}}$ at Air/2D/SiO_2_/Si and $\boldsymbol{r}$ at Air/SiO_2_/Si), can be obtained by solving unknown 2*n* equations for an *n*-layer system. The phase difference is defined as $\Delta\phi=\phi_{1}-\phi_{0}$, where $\phi_{0}$ = $Arg(\boldsymbol{r})$ and $\phi_{1}$ = $Arg\left( \boldsymbol{r}^{\boldsymbol{'}} \right)$. All calculations assume normal incidence (θ = 0).


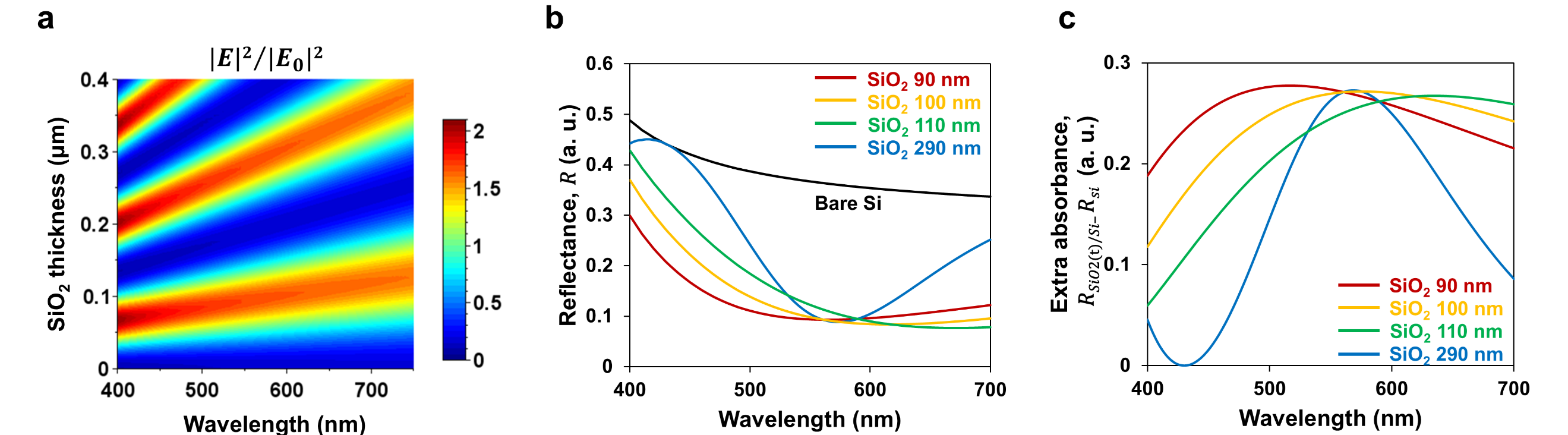


**Figure S4**. Theoretical calculation of three-layer system (air / SiO_2_ / Si). **(a)** Contour map of normalized electric field intensity as a function of SiO_2_ thickness and wavelength. **(b)** Reflectance spectra of bare Si substrate, 90, 100, 110, and 290 nm thick SiO_2_/Si substrate. **(c)** Extra absorbance ($\text{R}\text{SiO}\text{2(t)/}\text{Si}\text{–}\text{ }\text{R}\text{si}$, where $\text{R}\text{SiO}\text{2(t)/}\text{Si}$ is the reflectance from SiO_2_ /Si of with thickness t of SiO_2_ on Si substrate and $\text{R}\text{si}$ is the reflectance from bare Si substrate) spectra of 90–110 and 290 nm SiO_2_/Si substrate compared to the bare Si substrate.


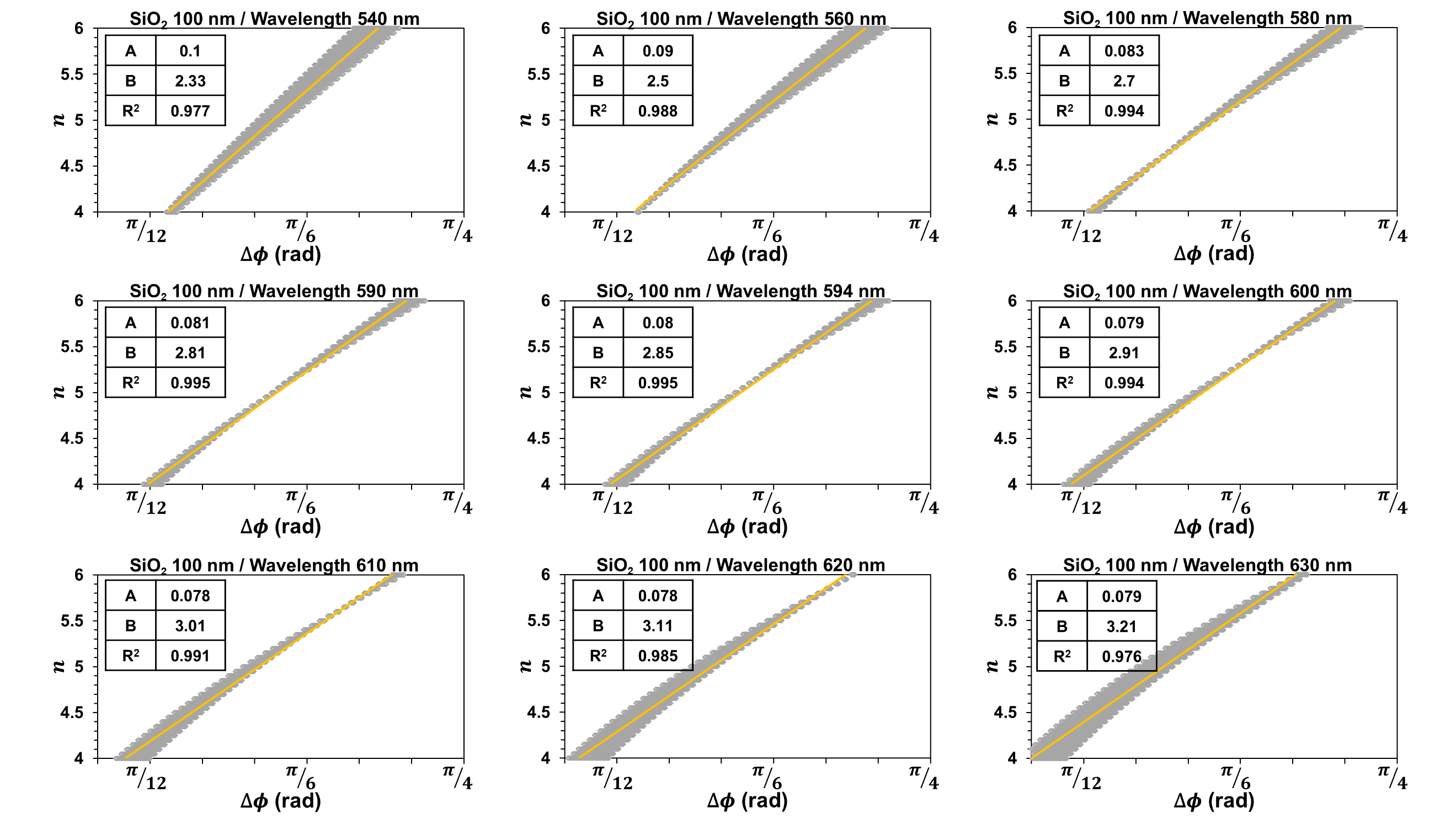
 **Figure S5**. Refractive index (*n*) scatter plots as a function of phase difference where *n* ranges 4–6, *k* varies 1–2, and 100 nm thick SiO_2_ is used. Orange trended lines from the linear regression were plotted with the chart of constants A and B, and R^2^ values.


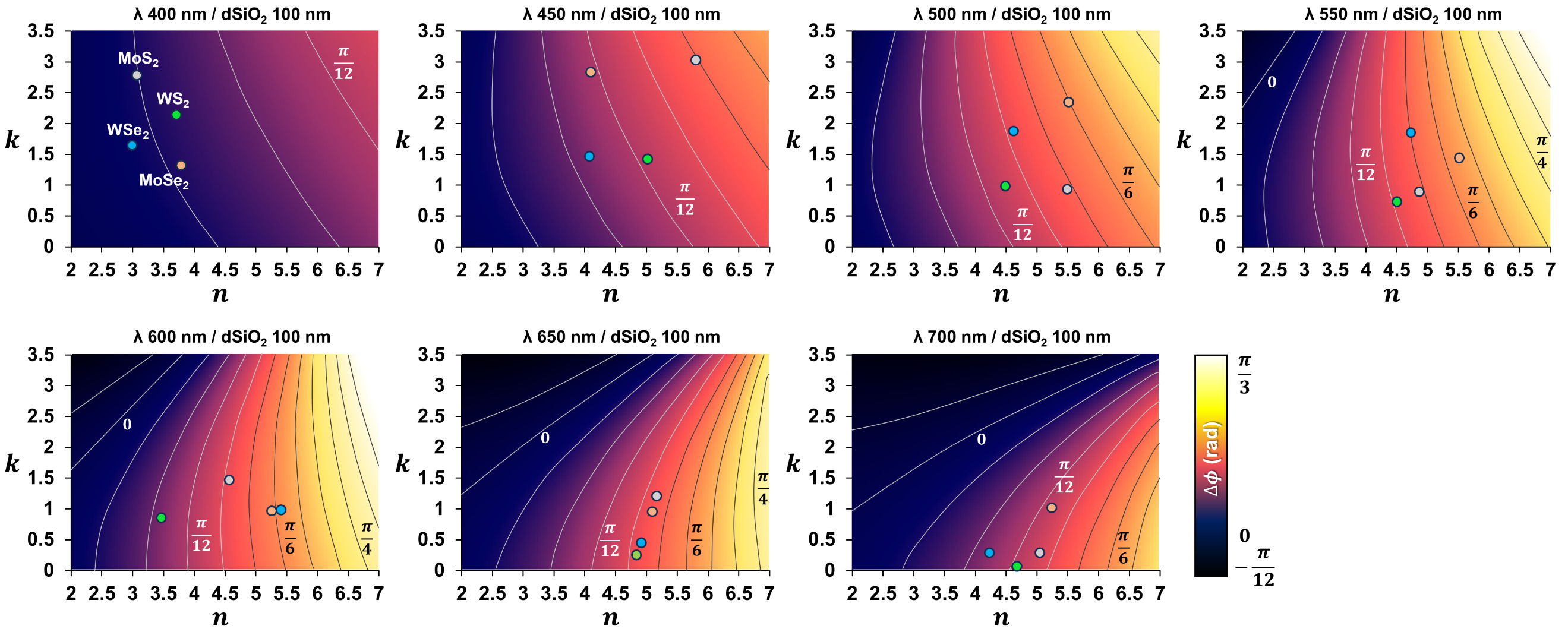


**Figure S6.** Contour maps of phase difference as a function of complex refractive indices (*n, k*) at a 100 nm thick SiO_2_/Si substrate and wavelength in the range of 400–700 nm at 50 nm intervals. Each colored dot represents the $n$ and $k$ values of four different kinds of transition metal dichalcogenides (MoS_2_, MoSe_2_, WS_2_ and WSe_2_) at each wavelength^[26]^.


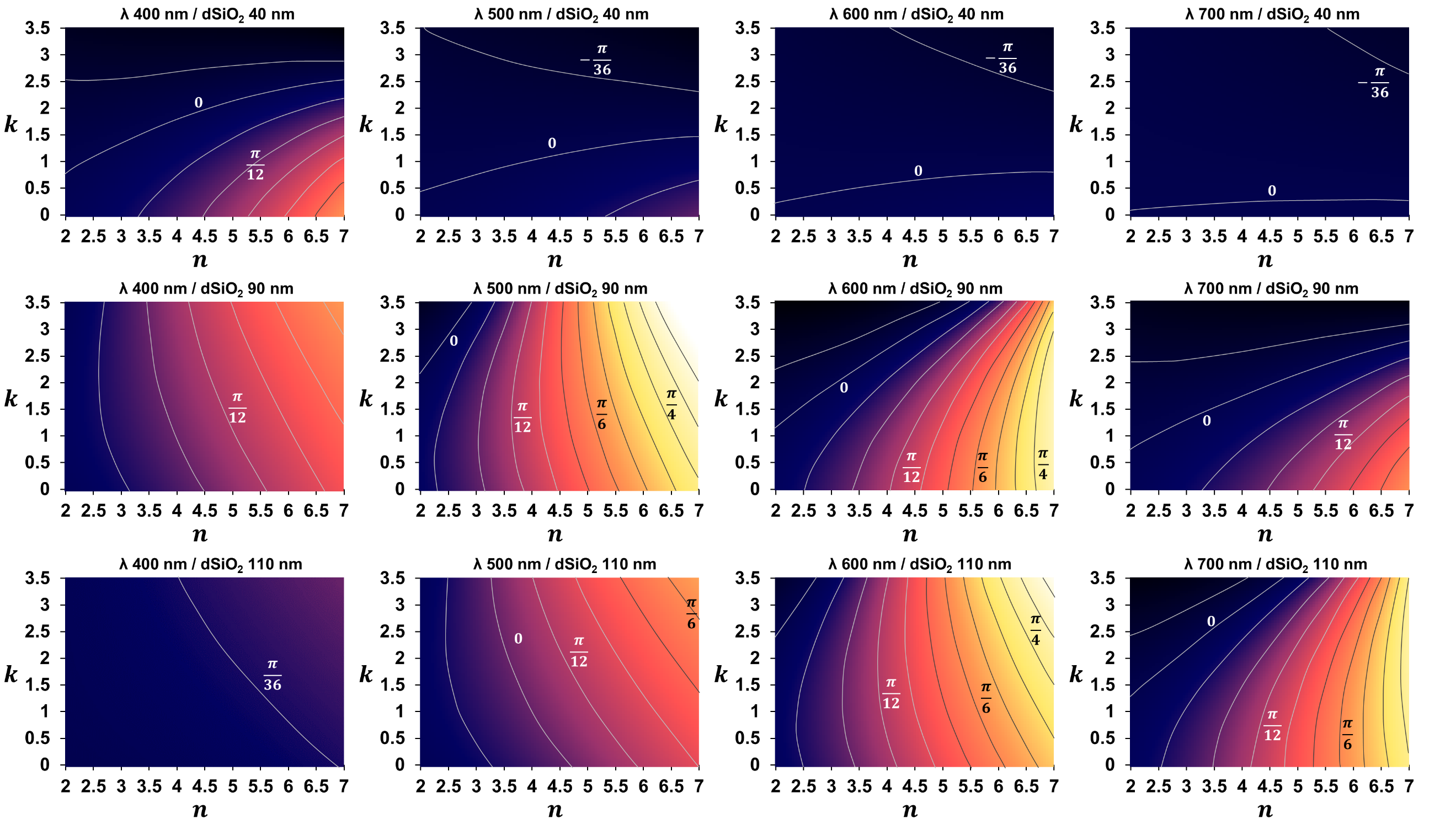

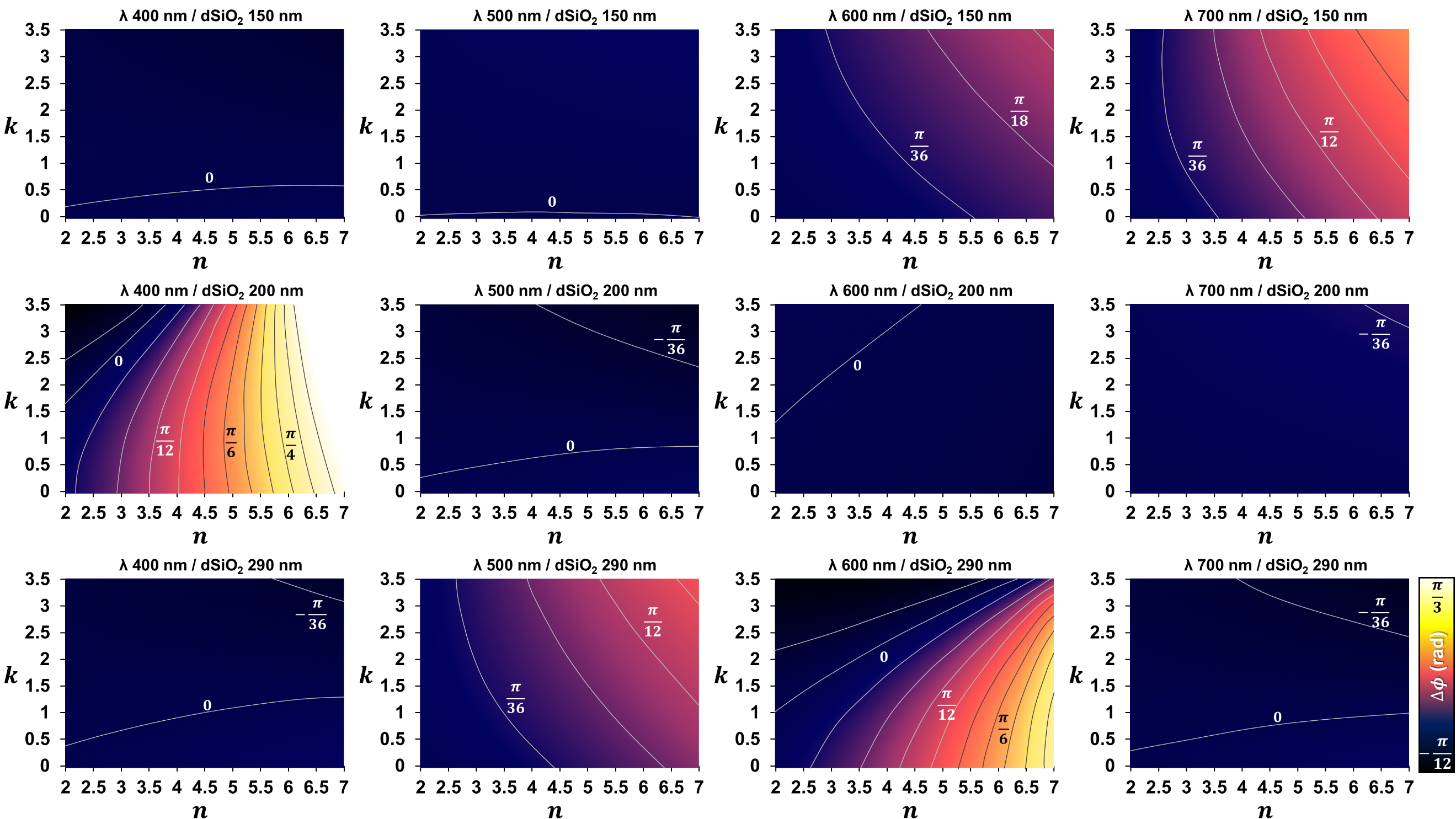
 **Figure S7.** Contour maps of phase difference as a function of complex refractive indices (*n, k*) at various SiO_2_ thicknesses and wavelength in the range of 400–700 nm at 100 nm intervals.

| **SiO_2_ thickness**  **(nm)** | **Wavelength**  **(**$\left\vert\frac{\boldsymbol{\partial(\Delta\phi)}}{\boldsymbol{\partial n}} \right\vert/\left\vert\frac{\boldsymbol{\partial(\Delta\phi)}}{\boldsymbol{\partial k}} \right\vert\boldsymbol{>3}$**)** | **Wavelength**  $\boldsymbol{(at}\frac{\boldsymbol{\partial(}\boldsymbol{\Delta\phi)}}{\boldsymbol{\partial k}}\boldsymbol{=0)}$ |
| --- | --- | --- |
| 10 ~ 60 | Out of visible range | - |
| 70 | 400 ~ 460 | 420 |
| 80 | 400 ~ 518 | 478 |
| 90 | 473 ~ 576 | 534 |
| 100 | 530 ~ 635 | 590 |
| 110 | 583 ~ 694 | 646 |
| 120 | 635 ~ 700 | 700 |
| 130 | 686 ~ 700 | > 700 |
| 140 ~ 190 | Out of visible range | - |
| 200 | 400 ~ 410 | < 400 |
| 210 | 400 ~ 430 | 416 |
| 220 | 410 ~ 450 | 436 |
| 230 | 433 ~ 468 | 456 |
| 240 | 454 ~ 488 | 474 |
| 250 | 472 ~ 508 | 494 |
| 260 | 492 ~ 526 | 514 |
| 270 | 512 ~ 546 | 532 |
| 280 | 530 ~ 566 | 552 |
| 290 | 550 ~ 585 | 570 |
| 300 | 568 ~ 604 | 590 |

**Table S1.** Wavelengths where $\left| \frac{\partial(\Delta\phi)}{\partial n} \right|/\left| \frac{\partial(\Delta\phi)}{\partial k} \right|>3$ and $\frac{\partial(\Delta\phi)}{\partial k}=0$ in dependence on SiO_2_ thickness, where MoS_2_ is used as the supported material. Partial derivatives $\frac{\partial(\Delta\phi)}{\partial n}$ and $\frac{\partial(\Delta\phi)}{\partial k}$ are calculated at *n* = 5 and *k* = 1.5.


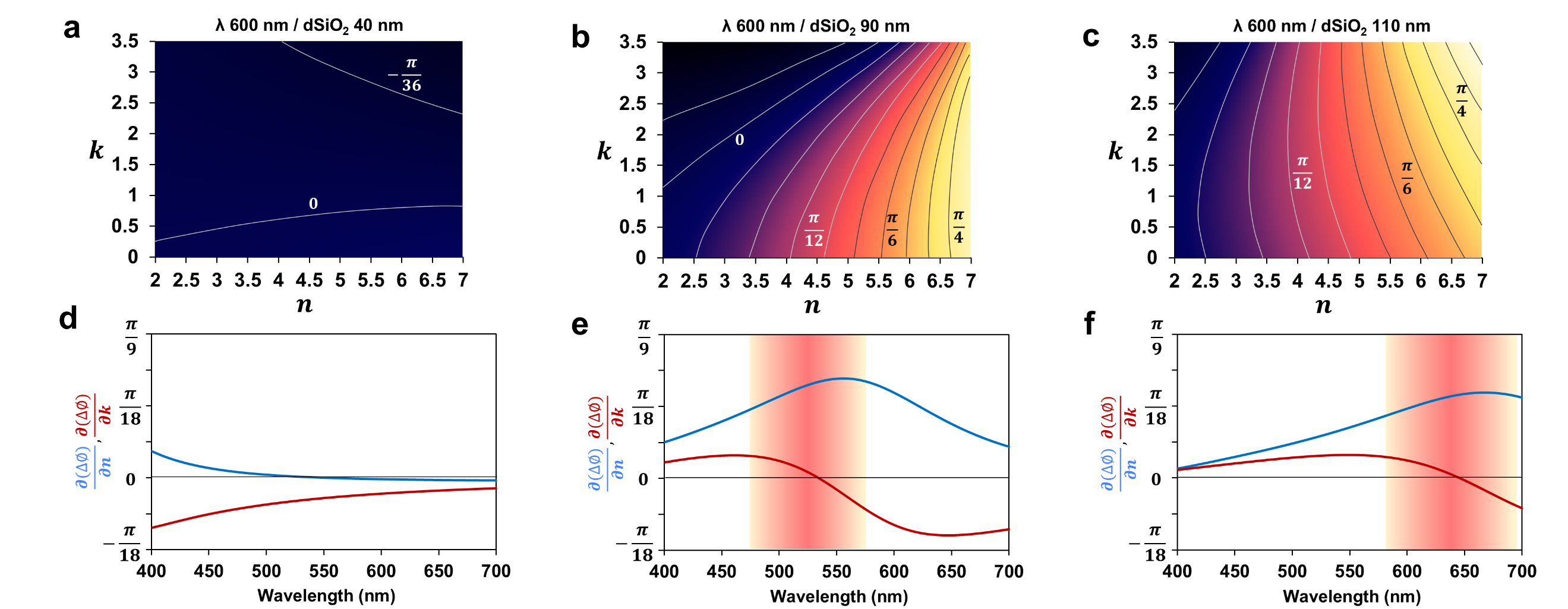

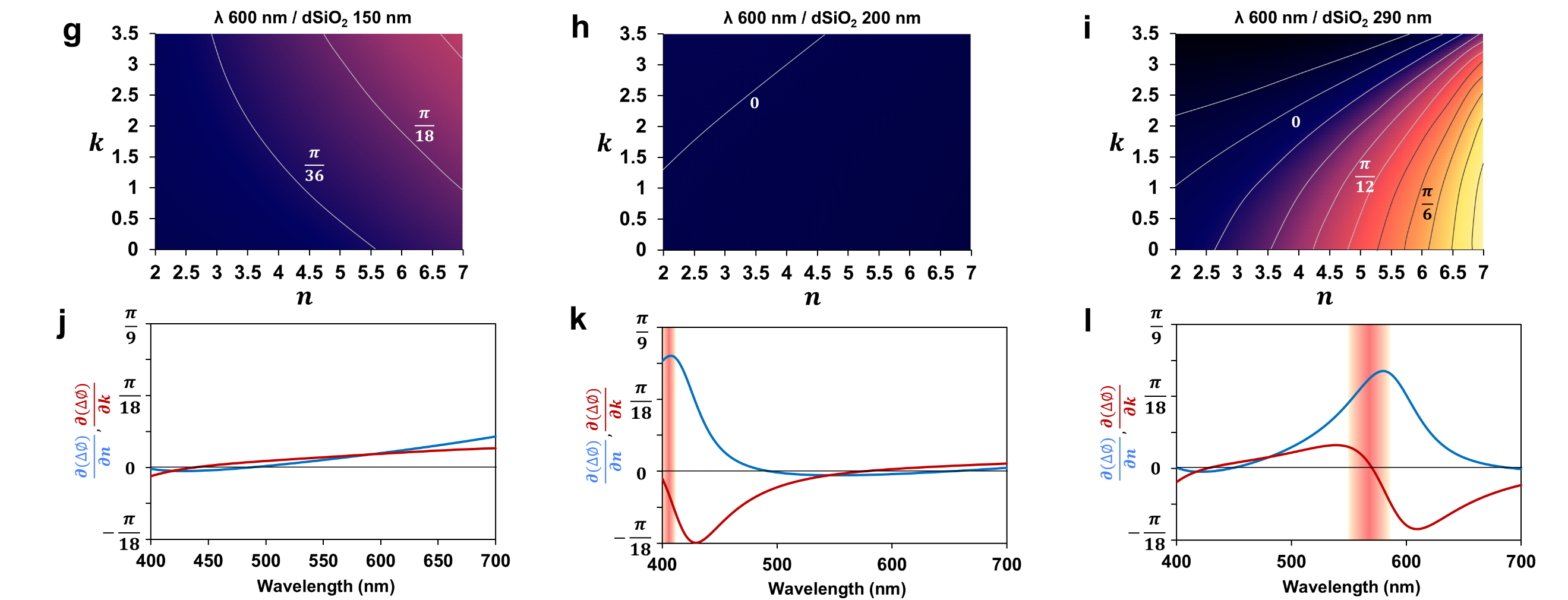


**Figure S8.** Contour maps and partial derivative spectra at various SiO_2_ thickness. **(a-c and g-i)** Contour maps of phase difference as a function of complex refractive indices (*n, k*) at wavelength of 600 nm. **(d-f and j-l)** Partial derivative spectra of phase difference to *n* and *k* ($\frac{\partial(\Delta\emptyset)}{\partial n}$, $\frac{\partial(\Delta\emptyset)}{\partial k}$) of each SiO_2_ thickness.

| **SiO_2_ thickness**  **(nm)** | **Wavelength (nm)** | $\boldsymbol{n=A*\Delta\phi+B}$ | | **R^2^** |
| --- | --- | --- | --- | --- |
|  |  | **A** | **B** |  |
| 100 nm | 500 | 0.129 | 2.08 | 0.953 |
|  | 510 | 0.12 | 2.13 | 0.959 |
|  | 520 | 0.113 | 2.19 | 0.965 |
|  | 530 | 0.106 | 2.25 | 0.971 |
|  | 540 | 0.1 | 2.33 | 0.977 |
|  | 550 | 0.095 | 2.41 | 0.983 |
|  | 560 | 0.09 | 2.5 | 0.988 |
|  | 570 | 0.086 | 2.6 | 0.991 |
|  | 580 | 0.083 | 2.7 | 0.994 |
|  | 590 | 0.081 | 2.81 | 0.995 |
|  | 594 | 0.08 | 2.85 | 0.995 |
|  | 600 | 0.079 | 2.91 | 0.994 |
|  | 610 | 0.078 | 3.01 | 0.991 |
|  | 620 | 0.078 | 3.11 | 0.985 |
|  | 630 | 0.079 | 3.21 | 0.976 |
|  | 640 | 0.081 | 3.3 | 0.965 |
|  | 650 | 0.083 | 3.39 | 0.950 |
|  | 660 | 0.086 | 3.47 | 0.931 |
|  | 670 | 0.09 | 3.55 | 0.910 |
|  | 680 | 0.094 | 3.63 | 0.886 |

**Table S2.** Constants A and B, and R^2^ values of the linear regression between phase difference and refractive index (*n*) with *n* ranging 4–6 and *k* ranging 1–2 when 100 nm thick SiO_2_ is used. The wavelengths of *n*-sensitive range ($\left| \frac{\partial(\Delta\phi)}{\partial n} \right|/\left| \frac{\partial(\Delta\phi)}{\partial k} \right|>3$) are gray-shaded.


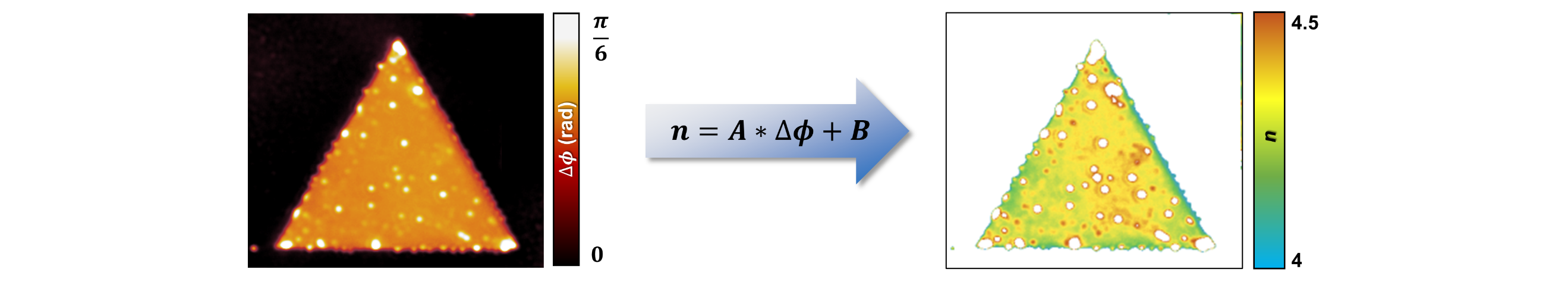


**Figure S9.** HPM image and constructed *n* distribution map at 589 nm of CVD as grown MoS_2_ supported on 98 nm SiO_2_/Si substrate.


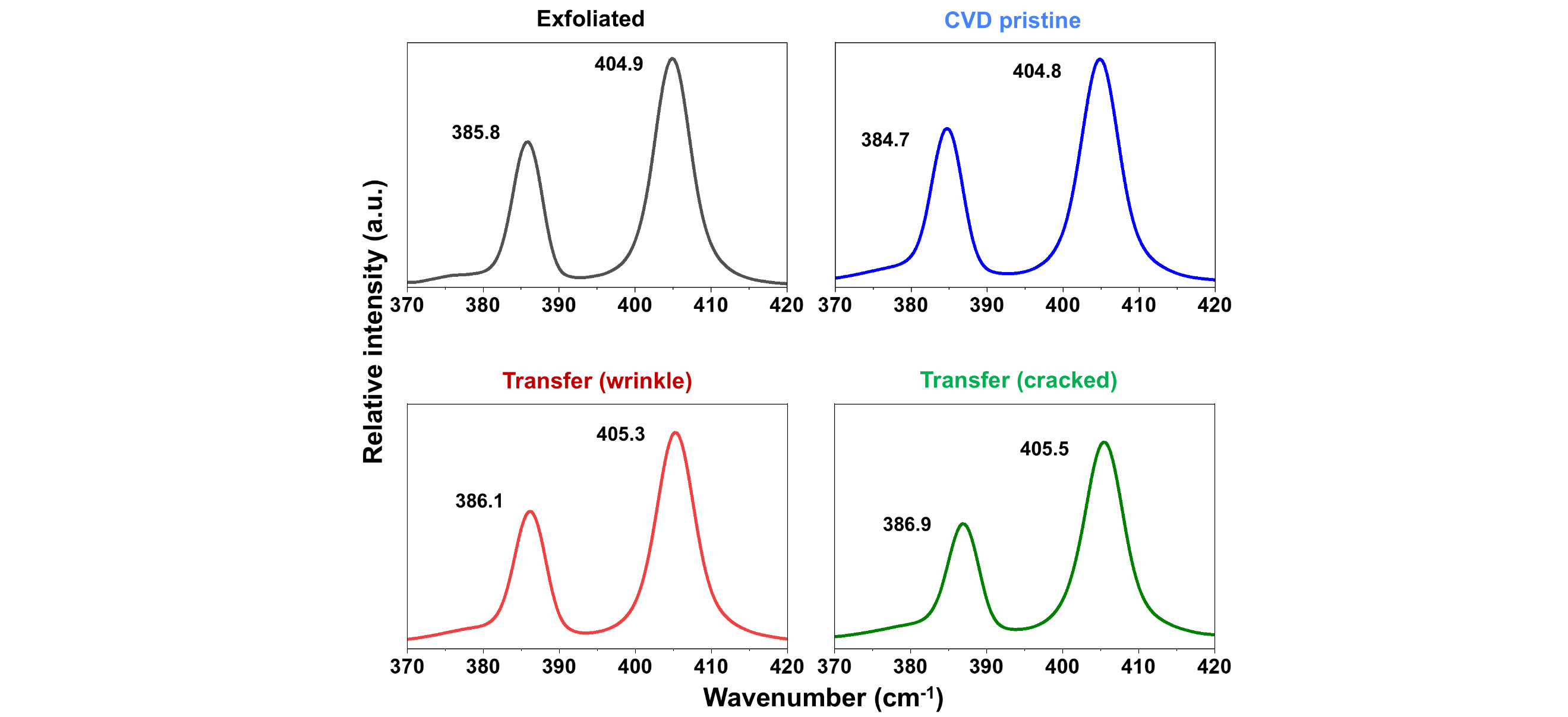


**Figure S10.** Averaged Raman spectra of MoS_2_ samples in **Figure 3a.**


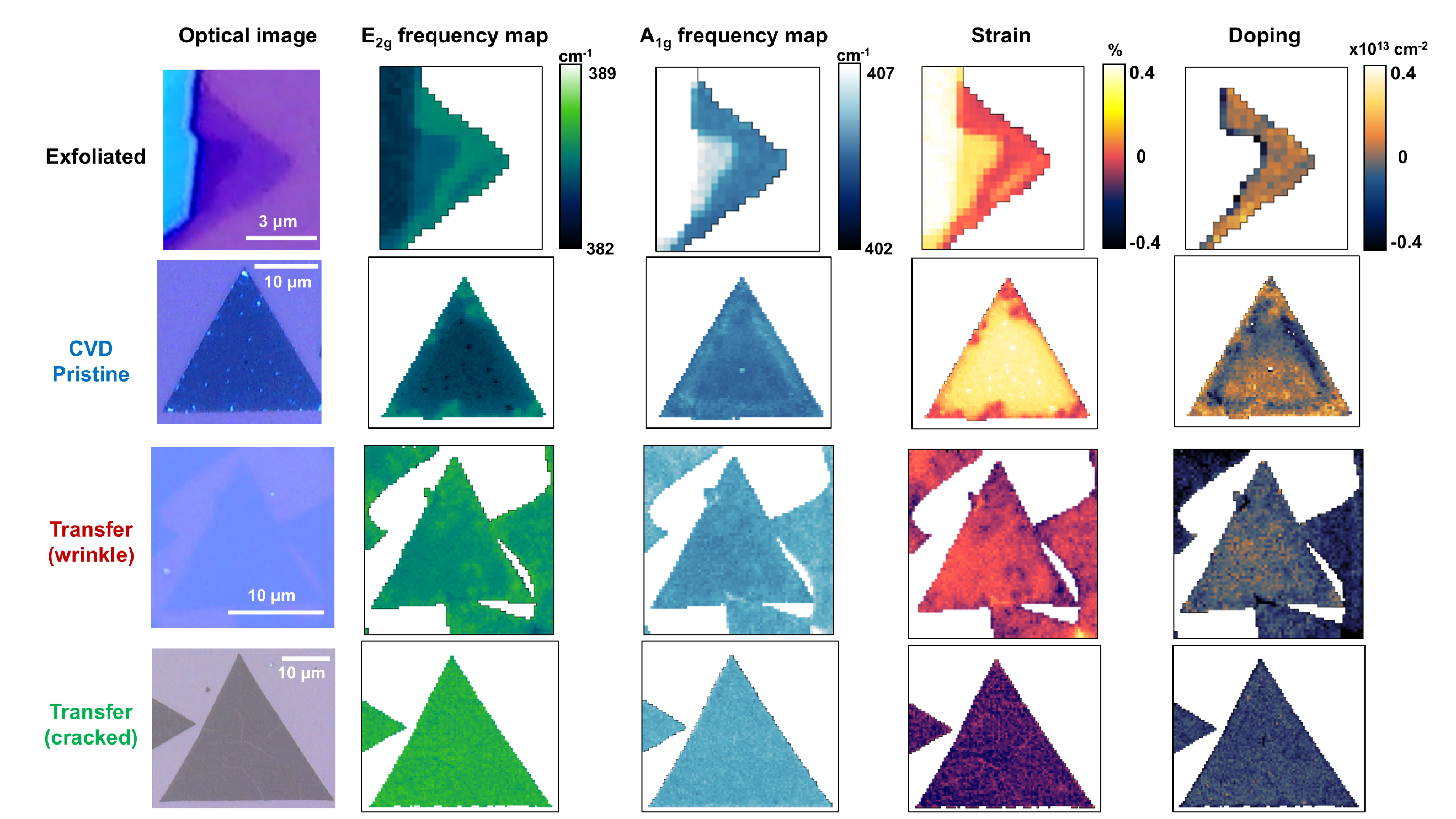


**Figure S11.** Optical images, Raman E_2g_ and A_1g_ frequency maps, strain, and doping maps of MoS_2_ samples in **Figure 3a**.


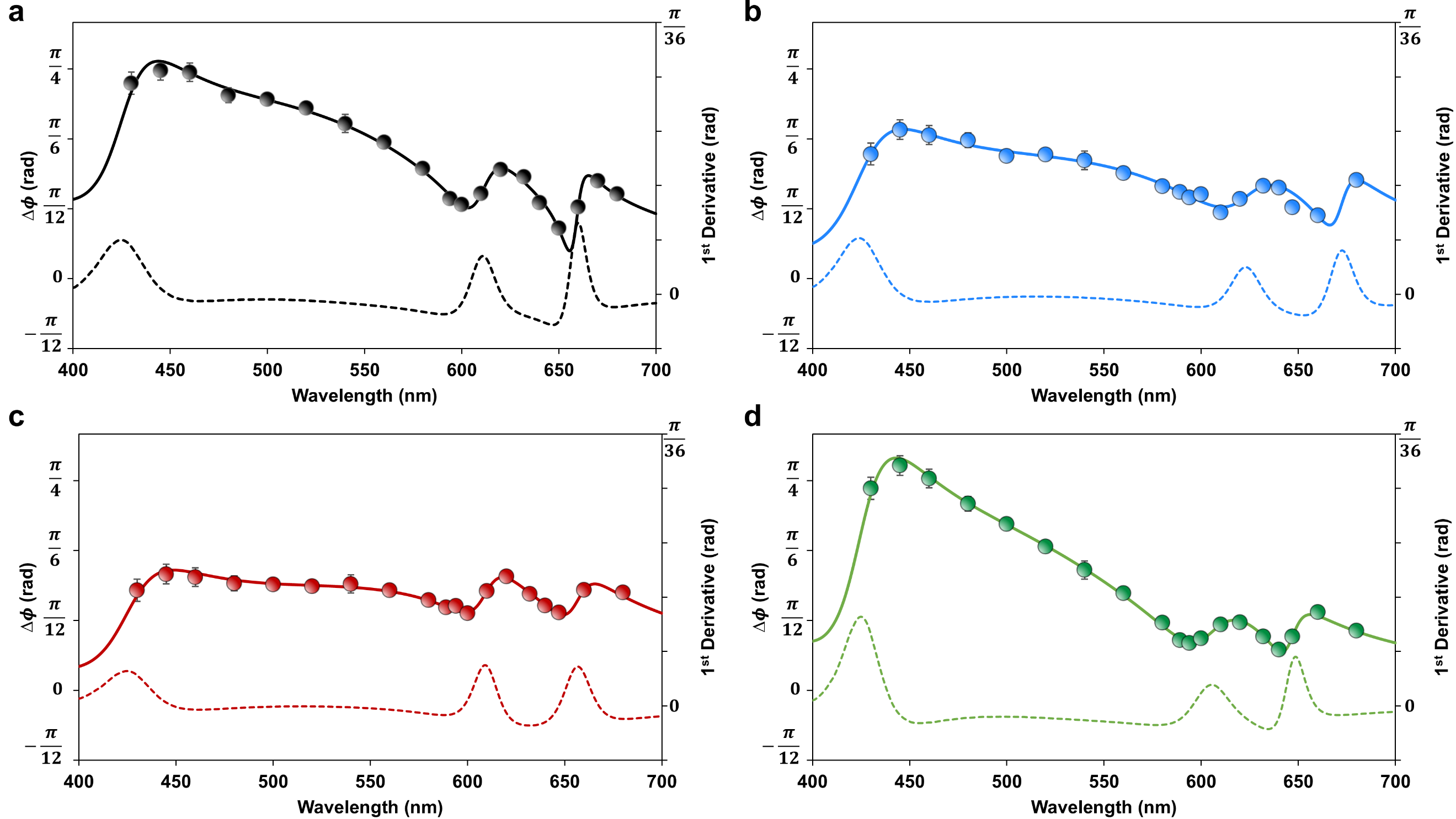
 **Figure S12.** Averaged phase differences measured by HPM (dots), fitted phase difference profiles using Lorentz oscillators model (solid lines) and first derivatives (dashed lines) of MoS_2_ samples in **Figure 3a**.


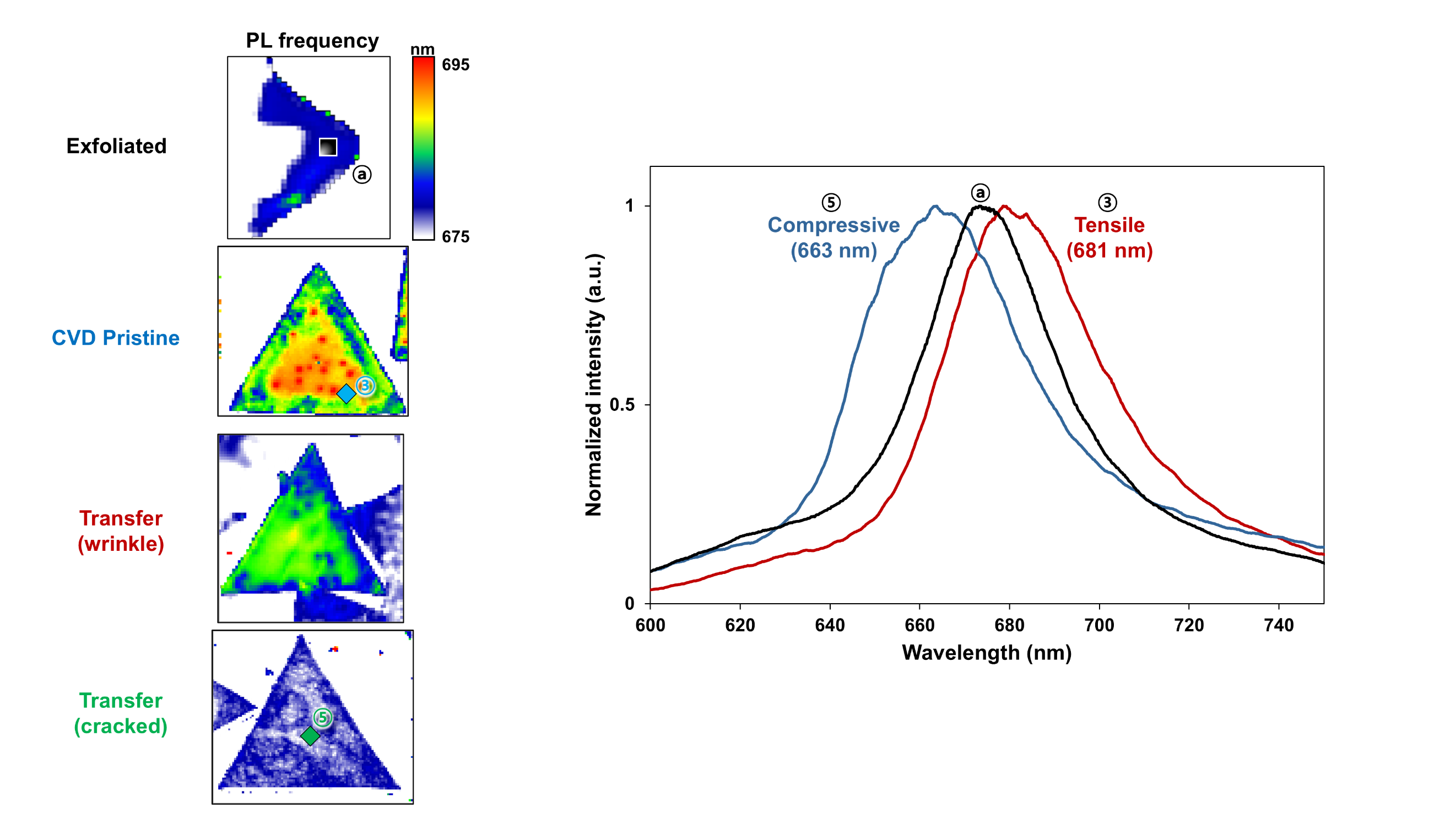
 **Figure S13.** PL frequency maps of MoS_2_ samples in **Figure 3a**, and normalized PL spectra without strain (black), with compressive (blue), and tensile (red) strain, respectively.


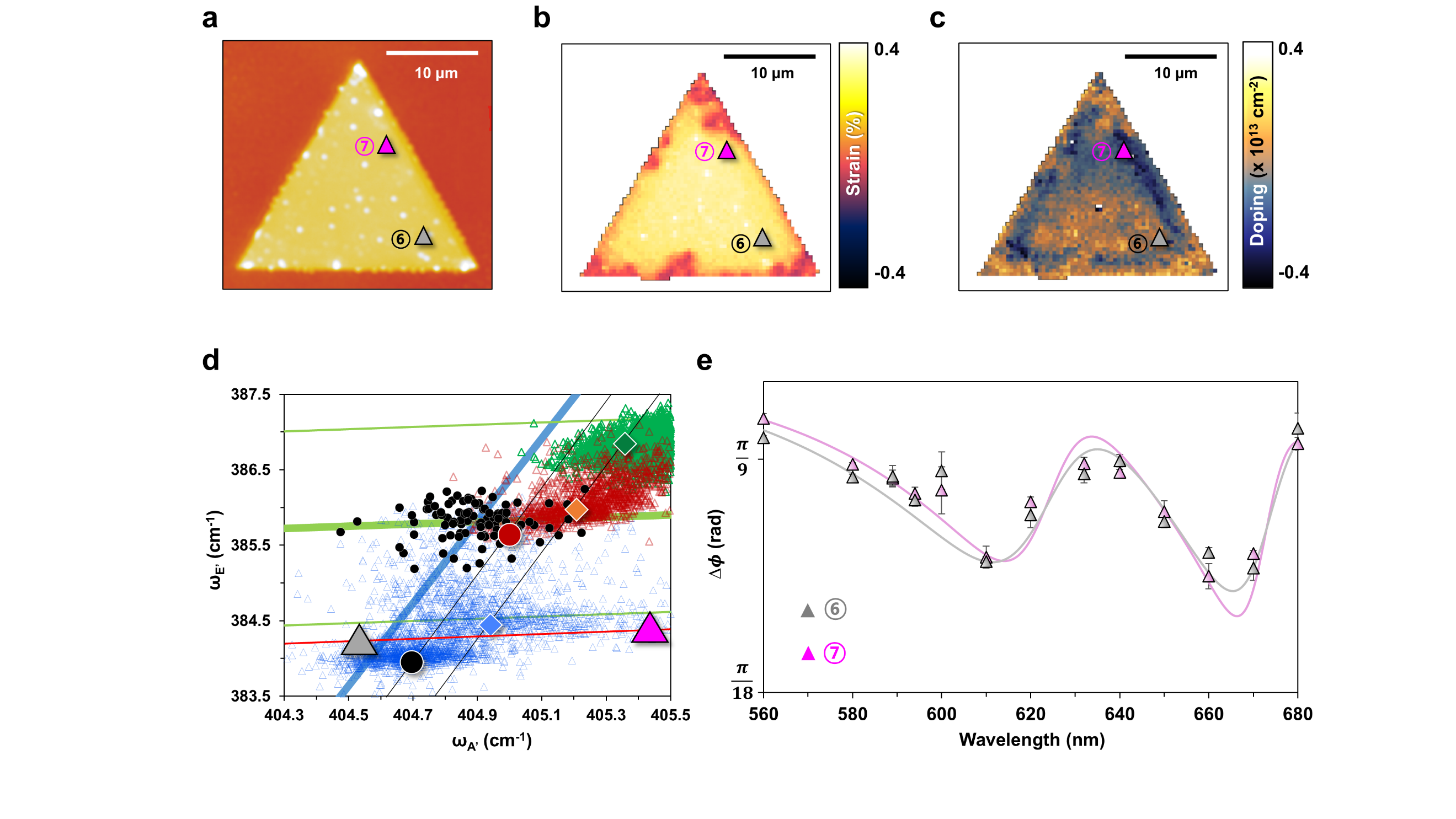


**Figure S14.** Study of phase difference for doping variation with strain fixed. **(a)** 540 nm phase map, **(b)** strain map, and **(c)** doping map of pristine MoS_2_ sample. **(d)** Blown-up Raman vector map from **Figure 3b** with marks of strain-independent points ⑥ and ⑦. (**e)** Phase difference profiles in the range of A- and B-excitons of strain-independent points.


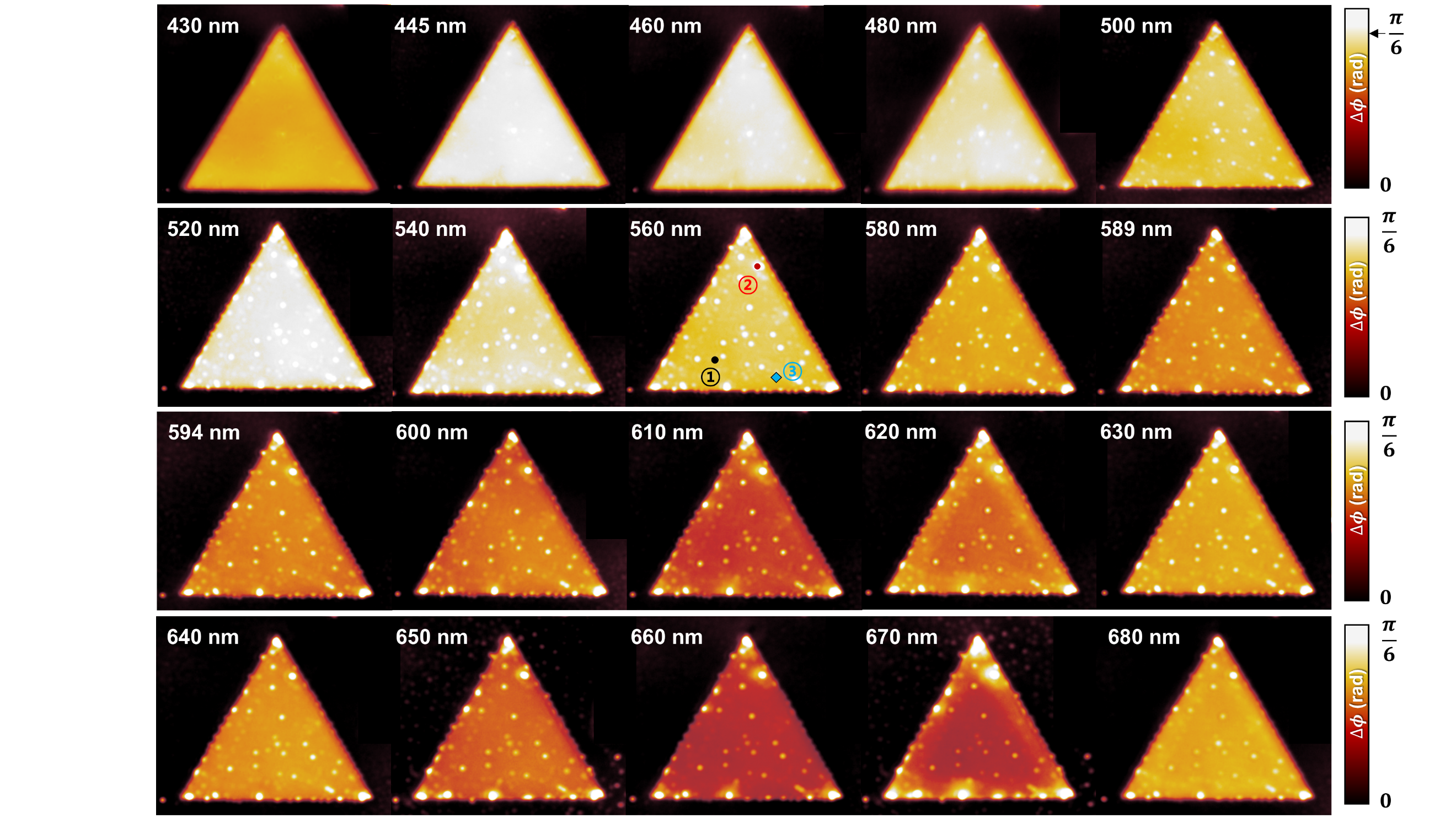


**Figure S15.** Phase difference images of CVD pristine MoS_2_ in the range of 430–680 nm.


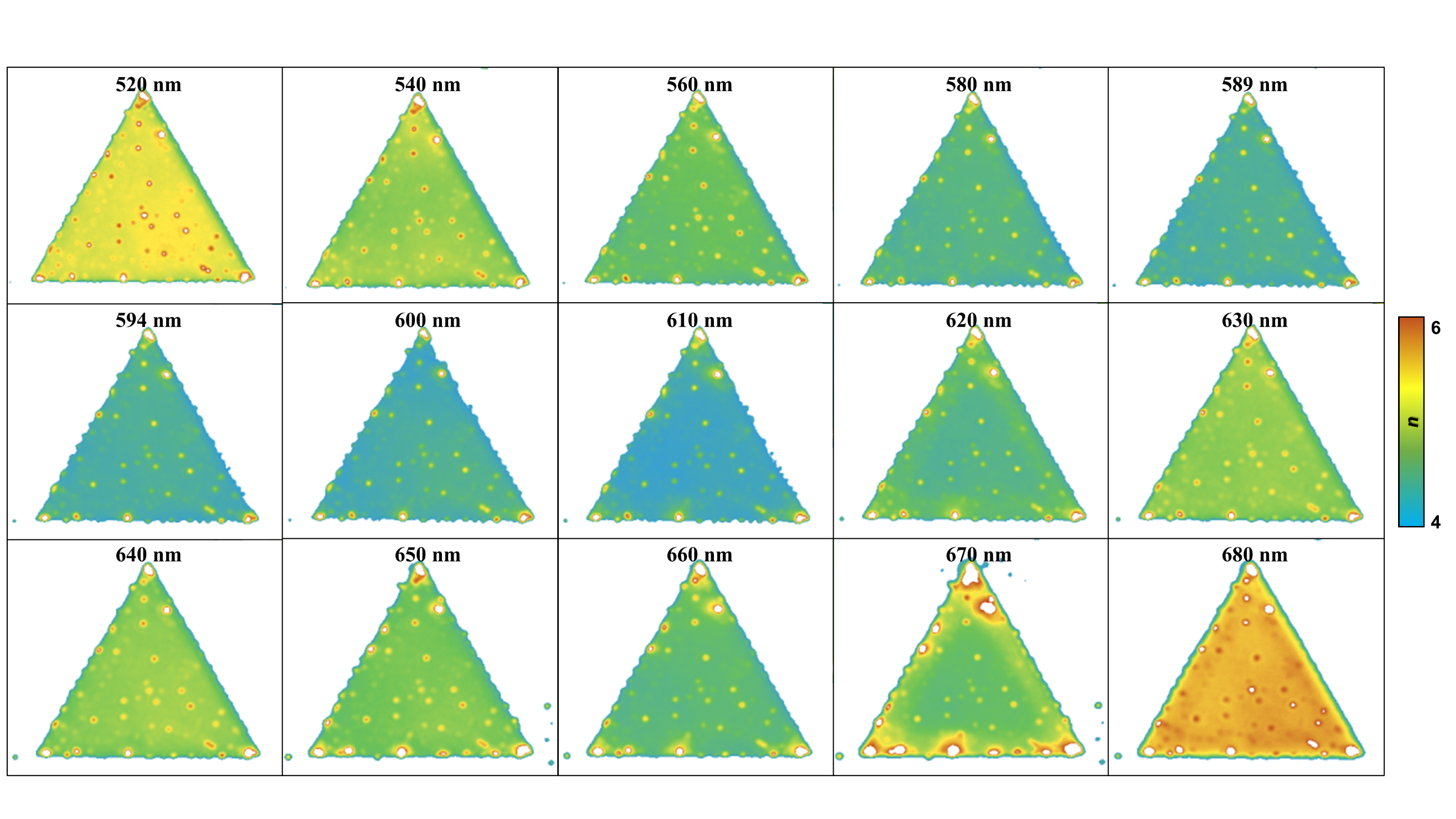


**Figure S16.** Spatially resolved refractive index (*n*) distribution maps constructed from phase difference images of CVD pristine MoS_2_ in the range of 520–680 nm.
